# Generation of Aerosols by Noninvasive Respiratory Support Modalities: A Systematic Review and Meta-Analysis

**DOI:** 10.1001/jamanetworkopen.2023.37258

**Published:** 2023-10-11

**Authors:** Madeline X. Zhang, Thijs A. Lilien, Faridi S. van Etten-Jamaludin, Carl-Johan Fraenkel, Daniel Bonn, Alexander P. J. Vlaar, Jakob Löndahl, Michael Klompas, Reinout A. Bem

**Affiliations:** 1 Institute of Physics, Van der Waals-Zeeman Institute, University of Amsterdam, Amsterdam, the Netherlands; 2 Department of Pediatric Intensive Care, Emma Children’s Hospital, Amsterdam UMC, University of Amsterdam, Amsterdam, the Netherlands; 3 Medical Library AMC, Amsterdam UMC, University of Amsterdam, Amsterdam, the Netherlands; 4 Division of Infection Medicine, Department of Clinical Sciences, Lund University, Lund, Sweden; 5 Department of Intensive Care, Amsterdam UMC, University of Amsterdam, Amsterdam, the Netherlands; 6 Division of Ergonomics and Aerosol Technology, Department of Design Sciences, Lund University, Lund, Sweden; 7 Department of Population Medicine, Harvard Medical School and Harvard Pilgrim Health Care Institute, Boston, Massachusetts; 8 Department of Medicine, Brigham and Women’s Hospital, Boston, Massachusetts; 9 Amsterdam Reproduction and Development Research Institute, Amsterdam, the Netherlands

## Abstract

**Importance:**

Infection control guidelines have historically classified high-flow nasal oxygen and noninvasive ventilation as aerosol-generating procedures that require specialized infection prevention and control measures.

**Objective:**

To evaluate the current evidence that high-flow nasal oxygen and noninvasive ventilation are associated with pathogen-laden aerosols and aerosol generation.

**Data Sources:**

A systematic search of EMBASE and PubMed/MEDLINE up to March 15, 2023, and CINAHL and ClinicalTrials.gov up to August 1, 2023, was performed.

**Study Selection:**

Observational and (quasi-)experimental studies of patients or healthy volunteers supported with high-flow nasal oxygen or noninvasive ventilation were selected.

**Data Extraction and Synthesis:**

Three reviewers were involved in independent study screening, assessment of risk of bias, and data extraction. Data from observational studies were pooled using a random-effects model at both sample and patient levels. Sensitivity analyses were performed to assess the influence of model choice.

**Main Outcomes and Measures:**

The main outcomes were the detection of pathogens in air samples and the quantity of aerosol particles.

**Results:**

Twenty-four studies were included, of which 12 involved measurements in patients and 15 in healthy volunteers. Five observational studies on SARS-CoV-2 detection in a total of 212 air samples during high-flow nasal oxygen in 152 patients with COVID-19 were pooled for meta-analysis. There was no association between high-flow nasal oxygen and pathogen-laden aerosols (odds ratios for positive samples, 0.73 [95% CI, 0.15-3.55] at the sample level and 0.80 [95% CI, 0.14-4.59] at the patient level). Two studies assessed SARS-CoV-2 detection during noninvasive ventilation (84 air samples from 72 patients). There was no association between noninvasive ventilation and pathogen-laden aerosols (odds ratios for positive samples, 0.38 [95% CI, 0.03-4.63] at the sample level and 0.43 [95% CI, 0.01-27.12] at the patient level). None of the studies in healthy volunteers reported clinically relevant increases in aerosol particle production by high-flow nasal oxygen or noninvasive ventilation.

**Conclusions and Relevance:**

This systematic review and meta-analysis found no association between high-flow nasal oxygen or noninvasive ventilation and increased airborne pathogen detection or aerosol generation. These findings argue against classifying high-flow nasal oxygen or noninvasive ventilation as aerosol-generating procedures or differentiating infection prevention and control practices for patients receiving these modalities.

## Key Points

**Question:**

Do high-flow nasal oxygen and noninvasive ventilation qualify as aerosol-generating procedures?

**Findings:**

This systematic review of 24 studies and meta-analysis of 5 studies found no evidence that either high-flow nasal oxygen or noninvasive ventilation results in clinically relevant increases in pathogen emission or aerosol production.

**Meaning:**

On the basis of the current evidence, high-flow nasal oxygen and noninvasive ventilation appear not to be aerosol-generating procedures and therefore do not merit differential infection prevention and control measures at this time.

## Teaser

This systematic review with meta-analysis evaluates current evidence to determine whether high-flow nasal oxygen and noninvasive ventilation are associated with pathogen-laden aerosols and aerosol generation.

## Introduction

During the recent worldwide SARS-CoV-2 outbreak, the potential harm for health care professionals working with infected patients received considerable attention.^1^ Within this context, there has been a special interest in the role of aerosols. Aerosols, consisting of liquid or solid airborne particles up to approximately 100 μm, have a much longer residence time in the air than larger (respiratory) droplets, which follow a ballistic trajectory under gravitational force. Aerosol emission from the respiratory tract of infected patients has been implicated as an important transmission route for SARS-CoV-2.^2,3,4^ In the early phase of the SARS-CoV-2 pandemic, infection prevention guidelines designated high-flow nasal oxygen (HFNO) and noninvasive ventilation (NIV) as possible aerosol-generating procedures (AGPs).^5,6,7^ This qualification suggested that these noninvasive respiratory support modalities might increase the risk of SARS-CoV-2 transmission to health care workers and therefore advised limiting their use, preferentially placing patients receiving HFNO or NIV in airborne infection isolation rooms and wearing a respirator rather than a medical mask when entering the room of patients with suspected or confirmed infection.^6,8,9,10^ The guidelines provided little or no data supporting the designation of HFNO and NIV as AGPs, however, which may have led to misdirected resources and confusion.^7,11,12,13,14,15^

Ideally, risk stratification for respiratory support modalities should be based on randomized clinical trials assessing associations between their use and health care worker infections. However, such studies have not been performed, and it is unlikely that they will be, given the complexity of randomizing critically ill patients and strained health care staff as well as the difficulty of attributing health care worker infections to specific exposures given the high prevalence of SARS-CoV-2 in the general community.^16^ Similarly, observational studies of the association between SARS-CoV-2 infections among health care workers and exposures to patients receiving HFNO or NIV are complicated by the difficulty of correcting for multiple potential confounders, including patient respiratory disease severity, viral load, quality of room ventilation, duration of health care worker exposure, proximity to patients, and alternative sources of health care worker exposure, including those outside the hospital.^17,18,19,20,21,22^

The best available evidence at this time to inform whether to classify HFNO and NIV as AGPs are studies quantifying pathogen-laden aerosols and aerosol production by HFNO and NIV. Several reviews on this topic have been published,^23,24,25^ but a systematic review with a quantitative synthesis of all experimental and observational aerosol collection studies is currently lacking. We sought to systematically review the literature of studies that investigate the potential for HFNO and NIV to increase pathogen-laden aerosols and aerosol production compared with treating patients without these modalities.

## Methods

### Data Sources and Searches

The literature search was performed in consultation with an experienced medical librarian (F.S.v.E.-J.). We searched the electronic databases PubMed/MEDLINE and Ovid EMBASE from inception to March 15, 2023, and the most recent systematic National Health Service rapid review^26^ for additional relevant articles. In addition, the CINAHL and ClinicalTrials.gov databases were queried on August 1, 2023. The complete and detailed search strategy is reported in the eMethods in Supplement 1. This review is reported in accordance with the Meta-analysis of Observational Studies in Epidemiology (MOOSE) reporting guideline^27^ and the Preferred Reporting Items for Systematic Reviews and Meta-Analyses (PRISMA) guidelines.^28^ The protocol of this review was registered before the start of the study in the PROSPERO database (CRD42023408378).

### Study Selection

The citations were screened independently by 2 reviewers (M.X.Z. and R.A.B.) on title and abstract, and discrepancies were resolved through discussion. Full-text articles were checked independently for eligibility. Eligible studies were those including patients or healthy volunteers receiving HFNO or NIV compared with unsupported normal or labored breathing, low-flow nasal oxygen (LFNO), or oxygen or nonrebreather mask (eMethods in Supplement 1). For studies using healthy volunteers fitted with HFNO or NIV, only studies reporting data from persons without a respirator (eg, N95) or surgical face mask were included. Both observational and experimental studies, including (quasi-)randomized controlled and crossover studies, were included. We excluded studies in which airborne particles were produced artificially by, for example, smoke generators or nebulization of chemical or salt solutions or that solely used computer modeling, because these studies cannot reliably determine actual human respiratory tract–derived aerosol particle production.

### Outcome Measures

The main outcomes were pathogen-containing aerosols (viable pathogen culturing from air specimens or DNA or RNA detection in air samples) and the number of aerosol particles (per time, air volume, or measurement unit or surrogate markers) smaller than 100 μm. Secondary outcomes were aerosol particle size distribution and pathogen detection in room surface samples.

### Data Extraction and Quality Assessment

Data were extracted independently by 3 authors (M.X.Z., T.A.L., and R.A.B.) using a structured form. Differences were resolved through discussion. Studies were grouped by patient or healthy volunteer and HFNO or NIV subgroups and summarized by main characteristics, outcome(s), and findings or study conclusions. Risk of bias was assessed as detailed in the eMethods in Supplement 1. Scoring was performed independently by 2 authors (T.A.L. and R.A.B.), and discrepancies were resolved through discussion.

### Statistical Analysis

For reporting main findings for each included study, the most relevant comparisons were summarized by medians (IQRs) or means (SDs) for continuous data. Because sample sizes of all studies were small, and thus likely subject to data skewness, no conversion of medians (IQRs) into means (SDs) was deemed appropriate. For dichotomous data, the studies are summarized using odds ratios (ORs) with 95% CIs or absolute findings.

For the main dichotomous outcome (pathogen detection in air sample) from the included observational studies, data were analyzed per support modality (HFNO or NIV) and compared with control (unsupported breathing, LFNO, or standard oxygen or nonrebreather mask). The data were analyzed by 2 definitions of event: (1) the number of pathogen-detected air samples of the total number of air samples and (2) the number of patients with at least 1 pathogen-detected air sample of the total number of patients to account for dependent data within treatment groups and between-study differences in the number of air samples collected per patient. We expected important between-study heterogeneity, primarily by design, together with sparse data and therefore used a random-effects Mantel-Haenszel model to pool all ORs in our primary analysis. Additional details, models, and post hoc sensitivity analyses are described in the eMethods in Supplement 1. The Paule-Mandel estimation was used to calculate heterogeneity variance τ^2^, as we did not anticipate large differences in sample sizes. Heterogeneity was further assessed by the *I*^2^ statistic, along with its uncertainty per 95% CI. A sensitivity analysis was performed by pooling ORs related to the number of patients with at least 1 pathogen-detected surface sample (secondary outcome) out of the total number of patients. Meta-analysis of continuous data (ie, aerosol particle concentrations) in experimental studies was deemed not appropriate for a combination of reasons, including inherent difficulties related to pooling of (nonrandomized) pre-post effect sizes,^29^ reported unit(s) and particle sizes of the outcome measurement, and reporting of medians (IQRs) based on skewed data. Publication bias was assessed by funnel plot. Significance was defined as a 2-sided *P* < .05. Analyses were performed using R software, version 4.2.1 (R Foundation for Statistical Computing) with RStudio, version 2022.02.3 + 492 (RStudio).

## Results

The database literature search resulted in 1735 potentially relevant studies. After screening, 24 studies^30,31,32,33,34,35,36,37,38,39,40,41,42,43,44,45,46,47,48,49,50,51,52,53^ remained (eFigure 1 in Supplement 1). Twelve studies^32,33,34,38,39,41,42,44,47,48,50,51^ investigated both HFNO and NIV, and 3 studies^33,40,53^ included both patients and healthy volunteers, using different study designs (see eTables 1, 3, and 4 in Supplement 1 for an overview and details of these studies). Overall, the sample size of the included studies was relatively small (range, 1-77 individuals), but otherwise risk of bias was deemed to be moderate to low (eMethods in Supplement 1). There was large variability in reporting of room conditions (eTable 2 in Supplement 1).

### High-Flow Nasal Oxygen

There were 2 (quasi-)experimental studies on HFNO that used a crossover design in adult patients; 1 randomized study focused on air and surface contamination of gram-negative bacteria in patients with gram-negative bacteria pneumonia,^30^ and 1 small study focused on aerosol particle concentration in patients with COVID-19.^31^ None of the studies found evidence of infectious airborne contamination or aerosol production by HFNO.

**Figure 1.**
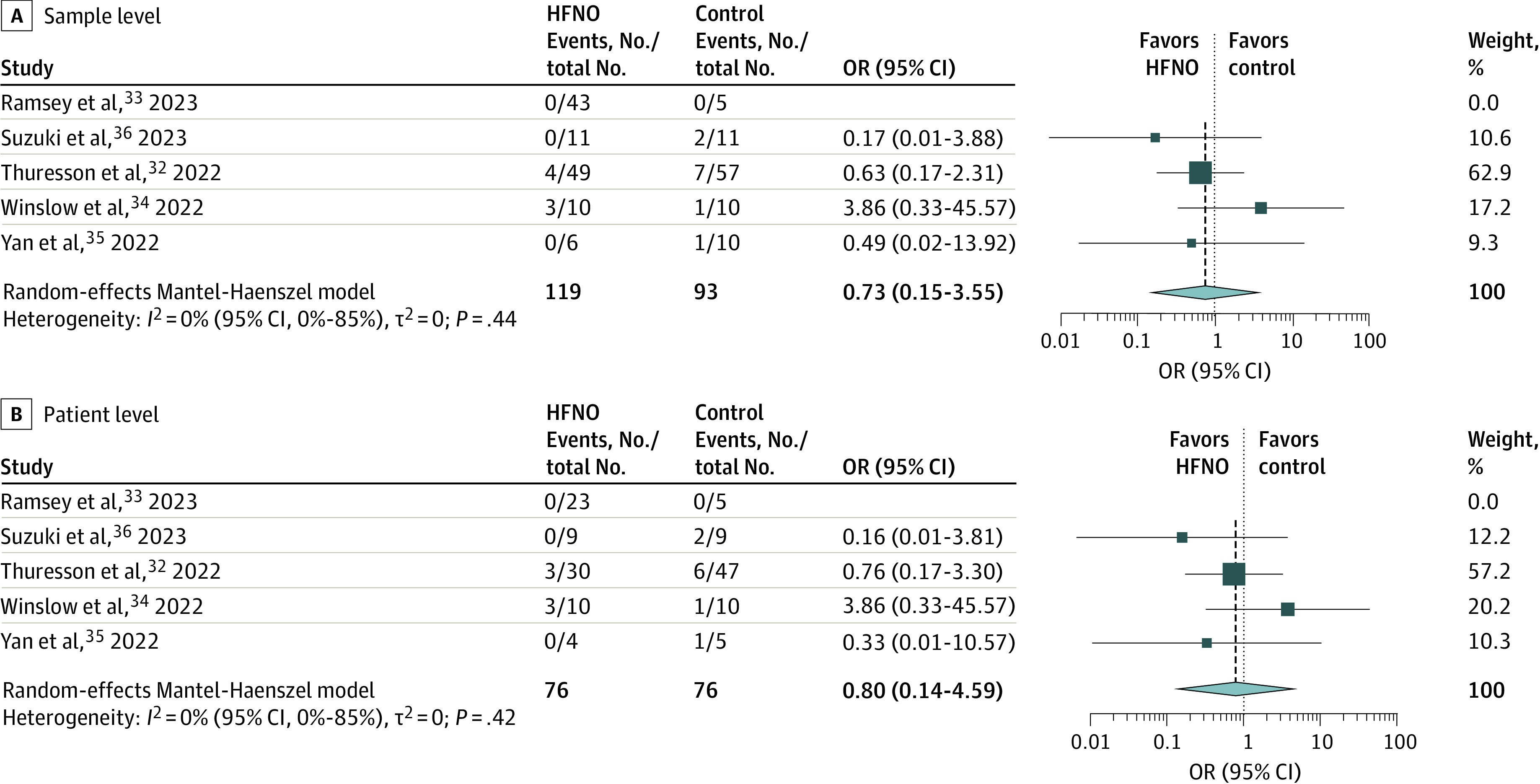
Random-Effects Meta-Analysis of High-Flow Nasal Oxygen (HFNO) on SARS-CoV-2 Detection in Air Samples From Patients With COVID-19 Forest plots showing the pooled odds ratios (ORs) and 95% CIs of observational studies assessing SARS-CoV-2 detection in air samples from patients with COVID-19 treated with either HFNO or control treatments (unsupported breathing, LFNO, or standard oxygen or nonrebreather mask). Patient-level data are from the study by Thuresson et al.^32^ Squares represent the relative weight of each study, and diamond size represents the summary effect size.

Of the 5 observational studies that investigated air samples for positive SARS-CoV-2 detection from patients with COVID-19,^32,33,34,35,36^ no study found evidence of an association between HFNO and increased airborne viral dispersion relative to unsupported breating, LFNO, or standard oxygen or nonrebreather mask. Meta-analysis of these studies, including a total of 212 air samples from 152 patients, did not show an association between HFNO and pathogen-containing air samples at either the sample level (OR, 0.73; 95% CI, 0.15-3.55; *P* = .58) or the patient level (OR, 0.80; 95% CI, 0.14-4.59; *P* = .71) (Figure 1). Model choice or double-zero studies did not influence outcome in the sensitivity analyses (eTable 5 in Supplement 1). The estimate of heterogeneity was highly uncertain for the sample- and patient-level analyses (*I*^2^ = 0%; 95% CI, 0%-85%); however, these studies^32,33,34,35,36^ differed substantially by air sampling methods and timing in relation to COVID-19 disease. Similarly, pooled sensitivity analysis of the studies that investigated potential surface contamination^34,37,38,39^ did not show an association between HFNO and pathogen-containing surface samples (eFigure 2 in Supplement 1). Finally, the single observational study that determined the number of aerosol particles surrounding patients without COVID-19 with acute severe respiratory illness found no association with HFNO treatment.^40^

There were 14 (quasi-)experimental studies involving aerosol particle detection in HFNO-treated healthy volunteers: 13 studies in adults^33,40,41,42,43,44,45,46,47,48,49,50,51^ and 1 in children.^52^ Of these, 9 studies found no statistically significant difference in the concentration of aerosols between HFNO and control treatments.^33,40,41,43,45,46,47,49,52^ In 1 study, no statistical analysis was performed because the data were derived from a single person.^51^ Four studies found a significant difference between the HFNO and the control groups.^42,44,48,50^ One of these studies found that increased aerosol concentration associated with HFNO was attributable to very small particles derived from the HFNO machine rather than from the study participants on further review.^44^ Of the remaining 3 studies, the effect size of the HFNO-induced increase in aerosol particle numbers was very small. For example, Wilson et al^50^ reported the largest effect: a 2.3-fold increase in particle counts associated with HFNO, but this was very small compared with a 371-fold increase induced by coughing alone without HFNO. One study, in which the investigators sampled air to detect an instilled chemical marker, found a small mean increase in volume for HFNO vs control (6.3 vs 0.0 μL/m^3^), but this corresponded to approximately 0.5% of the volume recovered during unsupported labored breathing and coughing.^46^

### Noninvasive Ventilation

**Figure 2.**
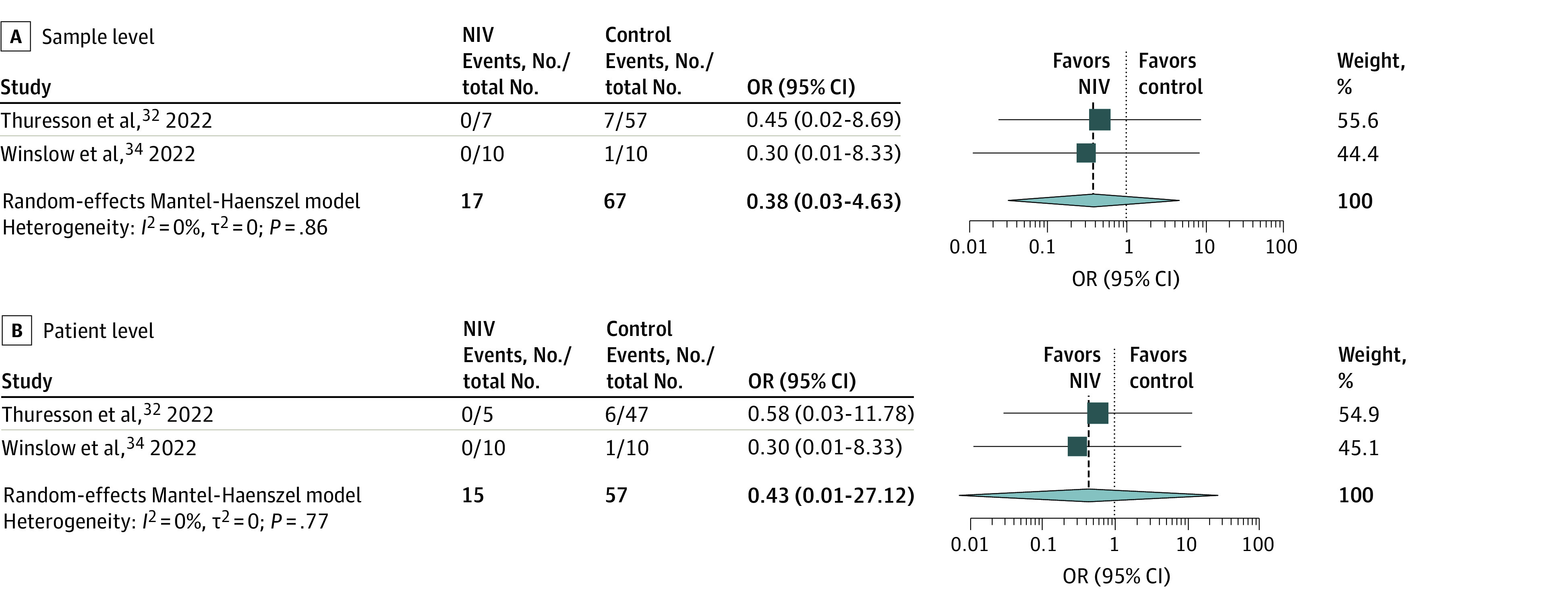
Random-Effects Meta-Analysis of Noninvasive Ventilation (NIV) on SARS-CoV-2 Detection in Air Samples From Patients With COVID-19 Forest plots showing the pooled odds ratios (ORs) and 95% CIs of observational studies assessing SARS-CoV-2 detection in air samples from patients with COVID-19 treated with NIV or control treatments (unsupported breathing, CFNO, or standard oxygen or nonrebreather mask). Patient-level data are from Thuresson et al.^32^ Squares represent the relative weight of each study, and diamond size represents the summary effect size.

The studies^32,33,34,38,39,41,42,44,47,48,50,51,53^ on NIV differed in use and level of bilevel or continuous positive pressure and use of vented (single limb) or nonvented (dual limb) face masks. Of the 2 observational studies that investigated air samples for positive SARS-CoV-2 detection from patients with COVID-19,^32,34^ neither found evidence of an association between NIV use and increased airborne viral dispersion. Meta-analysis of these studies, including a total of 84 air samples from 72 patients, also failed to show an association of NIV with pathogen-detected air samples either at the sample level (OR, 0.38; 95% CI, 0.03-4.63; *P* = .13) or at the patient level (OR, 0.43; 95% CI, 0.01-27.12; *P* = .24) (Figure 2). Again, model choice did not influence outcome (eTable 5 in Supplement 1). The uncertainty of heterogeneity could not be estimated based on the 2 studies. Similarly, pooled sensitivity analysis of the studies that investigated potential surface contamination^34,38,39^ did not show an association for NIV with pathogen-detected surface samples at the patient level (eFigure 2 in Supplement 1).

One nonrandomized study involving patients with coryza and acute-on-chronic respiratory disease showed a very small difference for aerosols 3 to 10 μm in vented NIV but not for nonvented NIV.^53^ For example, the mean difference in the number of particles larger than 10 μm per cubic meter was 0.666 to 0.807 particles for patients with NIV compared with unsupported breathing (*P* = .04), which seems very small when observing the mean difference of 1393 particles during a sequence of physiotherapy-assisted labored breathing and coughing. Furthermore, the study did not correct for multiple comparisons. There were 9 (quasi-)experimental studies assessing airborne particle detection around NIV-treated healthy volunteers.^33,41,42,44,47,48,50,51,53^ Of these, 7 studies found no statistically significant differences in the concentration of aerosols between NIV and control treatments.^33,41,42,44,47,48,53^ In 1 study, no statistical analysis was performed because the data were derived from a single individual.^51^ In the study by Wilson et al,^50^ a 2.6- to 7.8-fold increase in aerosol production by NIV was reported, but this effect was very small compared with a 371-fold increase by a coughing maneuver without NIV.

The GRADE (Grading of Recommendations, Assessment, Development, and Evaluations) summary of findings is presented in the Table. Publication bias was deemed unlikely based on funnel plotting (eFigure 3 in Supplement 1).

**Table.** Use of High-Flow Nasal Oxygen and Noninvasive Ventilation Compared With Unsupported Breathing or Conventional Oxygen Support

Primary outcome	Illustrative comparative risks per 1000 population^a^		Relative pooled effect, OR (95% CI)^b^	No. of samples or participants (No. of studies)	Certainty of the evidence (GRADE)	Comments
	Assumed risk with comparison	Corresponding risk with intervention (95% CI)				
**High-flow nasal oxygen**						
Pathogen-containing air sample (sample level)	118	89 (20-322)	0.73 (0.15-3.55)	212 (5)	Very low^c^	All studies included only patients with COVID-19.
Pathogen-containing air sample (patient level)	132	108 (21-411)	0.80 (0.14-4.59)	152 (5)	Very low^c^	
No. of aerosol particles	See comment	See comment	Not estimable	NA (15)	See comment	Assessment and description of aerosol counts was too heterogeneous among studies to allow direct comparison. See main text for narrative description of details.
**Noninvasive ventilation**						
Pathogen-containing air sample (sample level)	118	48 (4-382)	0.38 (0.03-4.63)	84 (2)	Very low^c^	All studies included only patients with COVID-19. No patients in the NIV group had a positive air sample.
Pathogen-containing air sample (patient level)	132	61 (2-805)	0.43 (0.01-27.12)	72 (2)	Very low^c^	
No. of aerosol particles	See comment	See comment	Not estimable	NA (9)	See comment	Assessment and description of aerosol counts was too heterogeneous among studies to allow direct comparison. See main text for narrative description of details.

Abbreviations: GRADE, Grading of Recommendations, Assessment, Development, and Evaluations; NA, not applicable; OR, odds ratio.

^a^ The assumed risk was based on the mean incidence of control groups of all studies. The corresponding risk (and its 95% CI) was based on the assumed risk and the OR of the intervention (and its 95% CI).

^b^ Random-effects model with Knapp-Hartung adjustment.

^c^ Downgraded because of imprecision of the CI and methodologic heterogeneity.

## Discussion

In this systematic review and meta-analysis, we identified 24 studies that investigated the potential of HFNO or NIV to increase pathogen-laden aerosols or aerosol generation from patients and healthy volunteers. Meta-analysis of observational studies did not show an association between these treatments and increased airborne pathogen detection. In addition, we found no convincing evidence from quasi-experimental studies that HFNO or NIV generates a clinically relevant (ie, likely to contribute to disease transmission) increase in aerosol particles relative to unsupported breathing alone or coughing.

In the current aftermath of COVID-19, the listing of HFNO and NIV as AGPs in national and international infection prevention guidelines is inconsistent. For example, the World Health Organization altered their listing during the COVID-19 pandemic based on evolving insights and has now deleted HFNO as a potential AGP, but NIV remains on their AGP list.^54^ This contrasts with the Centers for Disease Control and Prevention in the US, which currently lists NIV as an AGP and HFNO as a potential AGP.^55^ Finally, the National Health Service from the UK has omitted HFNO and NIV from their current guideline after their most recent (2022) rapid review, which stated that both treatments should be considered to be taken off the list.^26,56^ It has been advocated that the entire concept of AGPs needs to be abandoned.^24,25,57^ For example, personal protective equipment recommendations should be uniform for all suspected and infected patients, and room restrictions should be adapted on a patient-by-patient basis (eg, depending on viral load among other factors), but this approach may be difficult to implement.^25,57,58^

To our knowledge, this study is the most extensive systematic review with meta-analysis to date, and our findings corroborate previous reports on the topic.^23,26^ On meta-analysis, we found no evidence of an association between HFNO or NIV and increased airborne pathogen dispersion. There are no established standard procedures for sampling airborne pathogens. Therefore, data reporting is often incomplete, outcome measures vary, and detection limits are unclear, which makes comparisons between studies difficult. Likewise, no association between HFNO or NIV and increased surface contamination was found. However, surface contamination may be an imperfect measure of pathogen-laden aerosols because surfaces can be colonized by multiple routes and to varying degrees.

Importantly, the main respiratory physiologic mechanisms that explain aerosol emission from the human respiratory tract, including fluid film bursting in small airways and vibration of vocal cords,^59^ render HFNO and NIV less likely to be meaningful AGPs.^41^ Although the majority of studies from our review indeed found no increase in the concentration of aerosol particles by either HFNO or NIV, some studies found clinically questionable but statistically significant increases relative to unsupported breathing, LFNO, or oxygen mask. The reported effect sizes in these studies were very small, however, compared with increases in aerosol generation caused by labored breathing or coughing, suggesting that these symptoms are more reliable guides to aerosol production than respiratory support procedures per se.^40,44,46,50^

For this review, we excluded studies that investigated artificially derived particles (eg, by smoke generators) or that solely used computational models.^60,61,62,63,64^ This is because these studies cannot answer the question of whether HFNO or NIV creates a greater risk of generating aerosols from the human respiratory tract. Nevertheless, these studies add to the discussion about proximity and the risk of infectious disease transmission. For instance, it has been advocated that the increased flow velocity and continuous jet by HFNO or NIV lead to particle dispersion that is more widespread over longer distances.^25^ At the same time, these effects on risk of transmission by airflow would likely be highly similar to situations in which mechanical room ventilation, air conditioning, heat sources, or open windows cause air movements,^58^ which transport, disperse, and dilute any dense aerosol clouds from patients.

### Limitations

This study has some limitations, including small sample sizes from most of the included studies and a relative paucity of data on pathogens other than SARS-CoV-2. We were not able to perform a meta-analysis on the studies that investigated aerosol particle counts in crossover designs. Furthermore, there was high heterogeneity in the type of aerosol particle detectors, sampling methods, positions, and timing in relation to disease progression, as well as in reporting of units of aerosol measurements and size distribution. We also encountered poor descriptions of relevant experimental room conditions, most notably the number of air changes per hour, pressure settings, and relative humidity, which all influence aerosol dynamics and the accuracy of (optical) particle counters. This heterogeneity and lack of detail limits our conclusions and indicates the need for a more uniform approach in research on this topic. In addition, inclusion criteria for our search regarding the patient or volunteer category, (infectious) disease state, and control treatment were relatively broad, challenging the direct comparison and synthesis of studies. Finally, aerosol detection as well as measurement of pathogen DNA and RNA are imperfect surrogates for the human-to-human transmission risk of viable infectious particles.

## Conclusions

This systematic review and meta-analysis did not find evidence that either HFNO or NIV should be considered an AGP. Instead, the current literature suggests that both treatments do not increase pathogen-laden aerosols or aerosol generation at clinically relevant levels. Evidently, to avoid pathogen transmission, health care workers exposed to patients with respiratory infections should wear appropriate personal protection and manage their care in hospital room settings appropriate for specific pathogens; however, until further evidence arises, no differential protective measures based on exposure to HFNO- or NIV-treated patients are deemed necessary.

## Notes

Supplement 1. **eMethods.** Supplementary Methods **eFigure 1.** PRISMA Flow Diagram **eFigure 2.** Random Effects Meta-Analysis HFNO and NIV (Surface Samples) **eFigure 3.** Assessment of Publication Bias (Funnel Plots for Air Samples) **eTable 1.** Main Characteristics and Number of Identified Studies **eTable 2.** Reported Room Conditions **eTable 3.** Studies on HFNO **eTable 4.** Studies on NIV **eTable 5.** Summary of Sensitivity Analyses for HFNO and NIV Studies

Supplement 2.
Data Sharing Statement
