# Supplementary material for: Generation of Aerosols by Noninvasive Respiratory Support Modalities: A Systematic Review and Meta-Analysis
Source: JAMA Netw Open. 2023 Oct 11;6(10):e2337258. doi: 10.1001/jamanetworkopen.2023.37258 (PMC10568354; doi:10.1001/jamanetworkopen.2023.37258)
Supplement: Supplement 1. — eMethods. Supplementary Methods eFigure 1. PRISMA Flow Diagram eFigure 2. Random Effects Meta-Analysis HFNO and NIV (Surface Samples) eFigure 3. Assessment of Publication Bias (Funnel Plots for Air Samples) eTable 1. Main Characteristics and Number of Identified Studies eTable 2. Reported Room Conditions eTable 3. Studies on HFNO eTable 4. Studies on NIV eTable 5. Summary of Sensitivity Analyses for HFNO and NIV Studies [file jamanetwopen-e2337258-s001.pdf]

Supplementary Online Content

Zhang MX, Lilien TA, van Etten-Jamaludin FS, et al. Generation of aerosols by noninvasive respiratory support modalities: a systematic review and meta-analysis. *JAMA Netw Open*. 2023;6(10):e2337258. doi:10.1001/jamanetworkopen.2023.37258

eMethods. Supplementary Methods

eFigure 1. PRISMA Flow Diagram

eFigure 2. Random Effects Meta-Analysis HFNO and NIV (Surface Samples)

eFigure 3. Assessment of Publication Bias (Funnel Plots for Air Samples)

eTable 1. Main Characteristics and Number of Identified Studies

eTable 2. Reported Room Conditions

eTable 3. Studies on HFNO

eTable 4. Studies on NIV

eTable 5. Summary of Sensitivity Analyses for HFNO and NIV Studies

eReferences

This supplementary material has been provided by the authors to give readers additional information about their work.

© 2023 Zhang MX et al. *JAMA Network Open.*

eMethods. Supplementary Methods

Database search strategy

The databases PubMed/MEDLINE and Ovid EMBASE were searched from inception to March 15, 2023. Additionally, these searches were extended on August 1, 2023 with queries in CINAHL (Ebsco) and Clinicaltrials.gov. We checked all references of all identified articles. There was no language restriction. Preprints were excluded and conference abstracts were only included if sufficient data was provided to allow meta-analysis.

Databases:		
PubMed, Embase (Ovid), CINAHL (Ebsco), Clinicaltrials.gov	Before deduplication	After deduplication
Total	2194	1735

PUBMED

952 hits:

("Noninvasive Ventilation"[Mesh] OR "Respiratory Therapy"[Mesh] OR "Oxygen Inhalation Therapy"[Mesh] OR "Respiratory Therapy"[tiab] OR noninvasive ventilat*[tiab] OR non-invasive ventilat*[tiab] OR noninvasive mechanical ventilat*[tiab] OR non-invasive mechanical ventilat*[tiab] OR noninvasive respiratory support*[tiab] OR non-invasive respiratory support*[tiab] OR high flow nasal oxygen*[tiab] OR high flow nasal cannul*[tiab] OR high flow nasal therap*[tiab] OR high flow nasal prong therap*[tiab] OR HFNO[tiab] OR NIV[tiab] OR HF oxygen therap*[tiab] OR HFNC[tiab] OR oxygen therap*[tiab] OR oxygen treatment*[tiab] OR highflow nasal cannula[tiab] OR nasalhigh flow[tiab] OR nasal cannula therap*[tiab] OR nasal cannula noninvasive ventilation*[tiab] OR nasal cannula respiratory support[tiab] OR nasal prongs respiratory support[tiab] OR nasal prong therap*[tiab] OR nasal prongs therap*[tiab] OR nasal tube pulmonary ventilation*[tiab])

AND

("Respiratory Aerosols and Droplets"[Mesh] OR "Aerosolized Particles and Droplets"[Mesh] OR "Aerosols"[Mesh] OR aerosol*[tiab] OR droplet*[tiab] OR particle*[tiab] OR splatter*[tiab] OR microparticle*[tiab] OR microdroplet*[tiab] OR micro-particle*[tiab] OR micro-droplet*[tiab])

AND

("Infectious Disease Transmission, Patient-to-Professional"[Mesh] OR "Disease Transmission, Infectious"[Mesh] OR "transmission" [Subheading] OR "Infectious Disease"[tiab] OR "Viruses"[Mesh] OR "Virus Diseases"[Mesh] OR "Infections"[Mesh] OR "RNA, Viral"[Mesh] OR "Bacteria"[Mesh] OR spread*[tiab] OR superspread*[tiab] OR dispersion*[tiab] OR transmiss*[tiab] OR nosocomial*[tiab] OR infectious[tiab] OR infection*[tiab] OR virus*[tiab] OR SARS-CoV-2*[tiab] OR coronavirus*[tiab] OR COVID[tiab] OR COVID-19[tiab] OR cough*[tiab] OR sneez*[tiab] OR communicable disease*[tiab])

NOT

(("Animals"[MeSH Terms] OR "models, animal"[MeSH Terms] OR "Animal Experimentation"[MeSH Terms] OR rat[tiab] OR rats[tiab] OR mice[tiab] OR mouse[tiab] OR murine[tiab] OR murines[tiab] OR rodent[tiab] OR rodents[tiab] OR rabbit[tiab] OR rabbits[tiab] OR cat[tiab] OR cats[tiab] OR dog[tiab] OR dogs[tiab] OR pig[tiab] OR pigs[tiab] OR cow[tiab] OR cows[tiab] OR monkey[tiab] OR monkeys[tiab] OR goat[tiab] OR goats[tiab] OR horse[tiab] OR horses[tiab] OR ape[tiab] OR apes[tiab] OR gorilla[tiab] OR gorillas[tiab] OR sheep[tiab] OR sheeps[tiab] OR ovine[tiab] OR lamb[tiab] OR swine[tiab] OR swines[tiab] OR porcine[tiab] OR pup[tiab] OR pups[tiab] OR canine[tiab] OR beagle[tiab]) NOT "Humans"[MeSH Terms])

© 2023 Zhang MX et al. *JAMA Network Open.*

EMBASE (OVID):

Database(s): Embase Classic+Embase 1947 to 2023 March 14
Search Strategy:

#	Searches	Results
1	exp noninvasive ventilation/ or respiratory care/ or exp oxygen therapy/	114475
2	(noninvasive ventilat* or non-invasive ventilat* or noninvasive mechanical ventilat* or non-invasive mechanical ventilat* or noninvasive respiratory support* or non-invasive respiratory support* or high flow nasal oxygen* or high flow nasal cannul* or high flow nasal therap* or high flow nasal prong therap* or HFNO or NIV or HF oxygen therap* or HFNC or oxygen therap* or oxygen treatment* or highflow nasal cannula or nasalhigh flow or nasal cannula therap* or nasal cannula noninvasive ventilation* or nasal cannula respiratory support or nasal prongs respiratory support or nasal prong therap* or nasal prongs therap* or nasal tube pulmonary ventilation*).ti,ab,kf.	45995
3	1 or 2	131724
4	aerosol/ or aerosol generating procedure/	75143
5	(aerosol* or droplet* or particle* or splatter* or microparticle* or microdroplet* or micro-particle* or micro-droplet*).ti,ab,kf.	610707
6	4 or 5	632879
7	exp disease transmission/ or exp infection/ or exp virus/ or respiratory virus/ or virus RNA/ or exp bacterium/ or exp Coronavirus infection/	6376217
8	(spread* or superspread* or dispersion* or transmiss* or nosocomial* or infectious or infection* or virus* or SARS-CoV-2* or coronavirus* or COVID or COVID-19 or cough* or sneez* or communicable disease*).ti,ab,kf.	4155944
9	7 or 8	7604371
10	3 and 6 and 9	1128
11	(exp animal/ or exp animal experiment/ or exp animal model/ or exp veterinary medicine/ or (rat or rats or mice or mouse or murine or murines or rodent or rodents or rabbit or rabbits or cat or cats or dog or dogs or pig or pigs or cow or cows or monkey or monkeys or goat or goats or horse or horses or ape or apes or gorilla or gorillas or sheep or sheeps or ovine or lamb or swine or swines or porcine or pup or pups or canine or beagle).ti,ab,kw.) not human/	6793081
12	10 not 11	1091

CINAHL (Ebsco):

141 hits:

(MH "Oxygen Therapy" OR MH "Noninvasive Procedures" OR MH "Nasal Cannula") OR TI (noninvasive ventilat* OR non-invasive ventilat* OR noninvasive mechanical ventilat* OR non-invasive mechanical ventilat* OR noninvasive respiratory support* OR non-invasive respiratory support* OR high flow nasal oxygen* OR high flow nasal cannul* OR high flow nasal therap* OR high flow nasal prong therap* OR HFNO OR NIV OR HF oxygen therap* OR HFNC OR oxygen therap* OR oxygen treatment* OR highflow nasal cannula OR nasalhigh flow OR nasal cannula therap* OR nasal cannula noninvasive ventilation* OR nasal cannula respiratory support OR nasal prongs respiratory support OR nasal prong therap* OR nasal prongs therap* OR nasal tube pulmonary ventilation*) OR AB (noninvasive ventilat* OR non-invasive ventilat* OR noninvasive mechanical ventilat* OR non-invasive mechanical ventilat* OR noninvasive respiratory support* OR non-invasive respiratory support* OR high flow nasal oxygen* OR high flow nasal cannul* OR high flow nasal therap* OR high flow nasal prong therap* OR HFNO OR NIV OR HF oxygen therap* OR HFNC OR oxygen therap* OR oxygen treatment* OR highflow nasal cannula OR nasalhigh flow OR nasal cannula therap* OR nasal cannula noninvasive ventilation* OR nasal cannula respiratory support OR nasal prongs respiratory support OR nasal prong therap* OR nasal prongs therap* OR nasal tube pulmonary ventilation*)

AND

(MH "Aerosols" OR TI (aerosol* OR droplet* OR particle* OR splatter* OR microparticle* OR microdroplet* OR micro-particle* OR micro-droplet*) OR AB (aerosol* OR droplet* OR particle* OR splatter* OR microparticle* OR microdroplet* OR micro-particle* OR micro-droplet*))

AND

(MH "Virus Diseases+" OR MH "Disease Transmission" OR MH "COVID-19" OR (TI (spread* OR superspread* OR dispersion* OR transmiss* OR nosocomial* OR infectious OR infection* OR virus* OR SARS-CoV-2* OR coronavirus* OR COVID OR COVID-19 OR cough* OR sneez* OR communicable disease*) OR AB (spread* OR superspread* OR dispersion* OR transmiss* OR nosocomial* OR infectious OR infection* OR virus* OR SARS-CoV-2* OR coronavirus* OR COVID OR COVID-19 OR cough* OR sneez* OR communicable disease*)))

Clinicaltrials.gov

10 hits:

non invasive ventilation or respiratory care or oxygen therapy

AND

Aerosol

© 2023 Zhang MX et al. *JAMA Network Open.*

PICO

Participants/population

Patients (children and adults) with any bacterial or viral upper or lower respiratory tract infection in hospital wards, emergency departments or intensive care units, OR

healthy volunteer subjects (children and adults) in any type of (laboratory or hospital) setting.

Intervention(s), exposure(s)

Exposure to high flow nasal oxygen (HFNO) (warmed, humidified oxygen through nasal cannula with flow rate at 10-60 L/min for adults or > 1L/kg/min for children).

Exposure to non-invasive ventilation (NIV) (any continuous or bi-level positive pressure) with face mask by any design (vented/non-vented systems).

Comparator(s)/control

Unsupported normal/labored breathing, low flow nasal oxygen (LFNO) (by standard nasal cannula < 5L/min for adults or < 1L/kg/min for children), standard oxygen/non-rebreather mask (max. 15L/min), coughing, sneezing.

Outcome(s)

Pathogen-containing aerosols (viable pathogen culturing from air specimens or DNA/RNA detection in air samples) for patients and the number of aerosol particles (per time/air volume/measurement unit or surrogate markers) < 100µm for patients and healthy subjects. Secondary outcomes were aerosol particle size distribution and pathogen detection in room surface samples (for patients only).

© 2023 Zhang MX et al. *JAMA Network Open.*

Risk of bias assessment

For risk of bias assessment, we used the Revised Cochrane RoB2 tool for cross-over trials (version March 18, 2021)[2], the Newcastle-Ottawa Scale (NOS) for Cohort Studies for observational studies,[3] and the JBI Critical Appraisal Checklist for Quasi-Experimental studies for non-randomized cross-over studies.[4]

Note that some articles, which included more than one study design (e.g. both observational and experimental studies), have been assessed by two scoring systems.

Randomized experimental cross-over studies

Study	Domain 1: Randomization	Domain S: Period & Carry-over	Domain 2: Assignment intervention	Domain 3: Missing data	Domain 4: Measurement outcome	Domain 5: Reported results	Overall
**Jermy et al.[5]**	+/-	+	-	-	+/-	-	+/-
**Gaeckle et al.[6]**	+/-	+	-	-	-	-	+/-
**Leung et al.[7]**	-	+/-	-	-	-	-	-
**Miller et al.[8]**	-	+	-	-	-	-	+/-

By Revised Cochrane RoB2 tool for cross-over trials (version March 18, 2021). Rating for risk of bias: low: -; some concerns: +/-; high:+

Non-randomized experimental cross-over studies

Study	Q1: Cause-effect?	Q2: Similar participants?	Q3: Similar exposures?	Q4: Control group?	Q5: Multiple measurements[1] (timing)?	Q6: Follow-up complete?	Q7: Similar outcome measurement?	Q8: Reliable assessment?	Q9: Statistical analysis?[2]	Wash-out period?[3]	Overall
**Bem et al.[9]**	y	y	y	y	y	y	y	y	y	y	-
**Gall et al.[10]**	y	y	y	y	y	y	y	y	n	y	+/-
**Hamada et al.[11]**	y	y	y	y	y	y	y	y	n	u	+/-
**Hamilton et al.[12]**	y	y	y	y	y	y	y	y	y	u	+/-
**Helgeson et al.[13]**	y	y	y	y	y	y	y	y	n	u	+/-
**Li et al.[14]**	y	y	y	y	y	y	y	y	u	u	+/-
**McGain et al.[15]**	y	y	y	y	y	y	y	y	n	y	+/-
**Pearce et al.[16]**	y	y	y	y	y	y	y	y	y	y	-
**Ramsey et al.[17]**	y	y	y	y	y	y	y	y	y	y	-
**Simonds et al.[18]**	y	y	y	y	y	y	y	y	y	y	-
**Strand-Amundsen et al.[19]**	y	y	y	y	y	y	y	y	y	y	-
**Takazono et al.[20]**	y	y	y	y	y	y	y	y	y	u	+/-
**Wilson et al.[21]**	y	y	y	y	y	y	y	y	y	y	-

By JBI critical appraisal tool for Quasi-Experimental (non-randomized) studies. Answers: yes (y), no (no), unlcear (u), not applicable (na). Overall rating: low: -; some concerns: +/-; high:+. [1]continuous measurements were

© 2023 Zhang MX et al. *JAMA Network Open.*

performed for a standard period during exposure/control; [2]appropriate is defined as paired/repeated measures/baseline normalized analysis for cross-over design. [3]Additionally, these studies were checked for including a reported wash-out period to avoid carry-over effects of the cross-over treatments.

Observational studies

Study	Selection				Comparability		Outcome			Total score
	Representativeness	Selection of non-exposed cohort	Ascertainment of exposure	Outcome not present at start study	Most important factor	Any additional factor	Assessment outcome	Follow-up length	Follow-up adequacy	
**Bem et al.[9]**	*	*	*	*			*	*	*	7/9
**Lomont et al.[22]**	*	*	*		*		*	*	*	7/9
**Mendes et al.[23]**		*	*				*	*	*	5/9
**Ong et al.[24]**	*	*	*		*		*	*	*	7/9
**Ramsey et al.[17]**	*	*	*		*		*	*	*	7/9
**Suzuki et al.[25]**	*	*	*		*		*	*	*	7/9
**Thuresson et al.[26]**	*	*	*		*	*	*	*	*	8/9
**Winslow et al.[27]**	*	*	*		*		*	*	*	7/9
**Yan et al.[28]**	*	*	*				*	*	*	6/9

By the Newcastle-Ottowa Quality Assessment Scale for cohort studies. Total score: <4: high, <7: moderate, 7-9: low risk of bias.

Additional statistical analysis

The primary analysis consisted of the random-effects Mantel-Haenszel (MH) model to pool all ORs in. However, random-effects models may potentially result in biased effect estimates when events are rare, especially in the presence of heterogeneity.[29] Knapp-Hartung adjustment of the pooled CI was therefore applied to account for the latter and the low number of studies.[30] Additionally, we used a ‘true’ fixed-effect MH model given its improved performance under sparse data and to assess the influence of model choice.[31] Continuity correction was not applied, unless all studies included 0 events in the same exposure group, in such cases 0.5 was added to all cells (Haldane-Anscombe correction). A post-hoc analysis of the arcsine difference was added to assess the influence of double-zero studies on the outcome.

Assessment of publication bias

Publication bias was deemed unlikely since all studies included in the pooled analysis reported negative results as also observed in the funnel plot (eFigure 3).

© 2023 Zhang MX et al. *JAMA Network Open.*

eFigure 1. PRISMA Flowchart of Study Screening Process

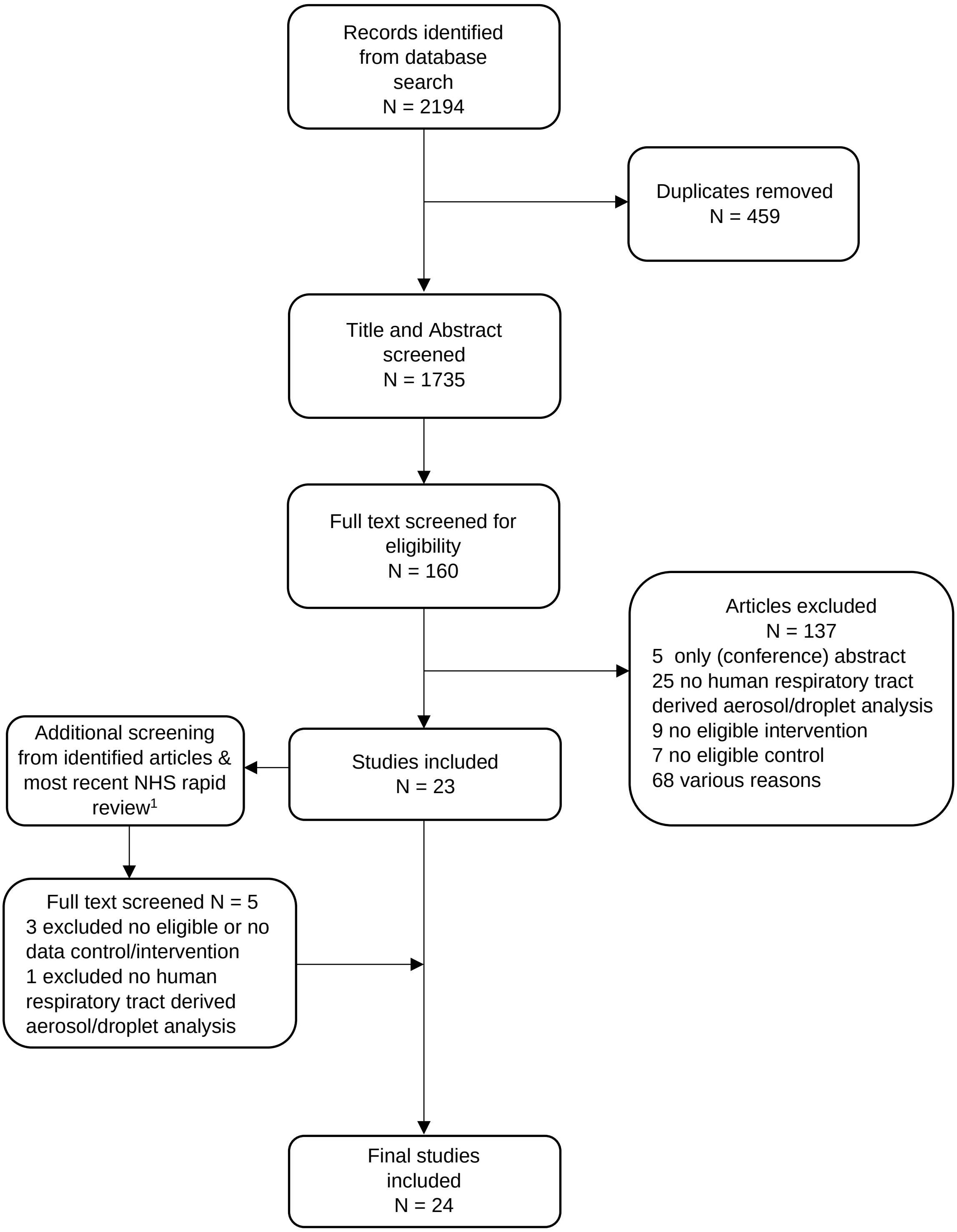

© 2023 Zhang MX et al. *JAMA Network Open.*

eFigure 2

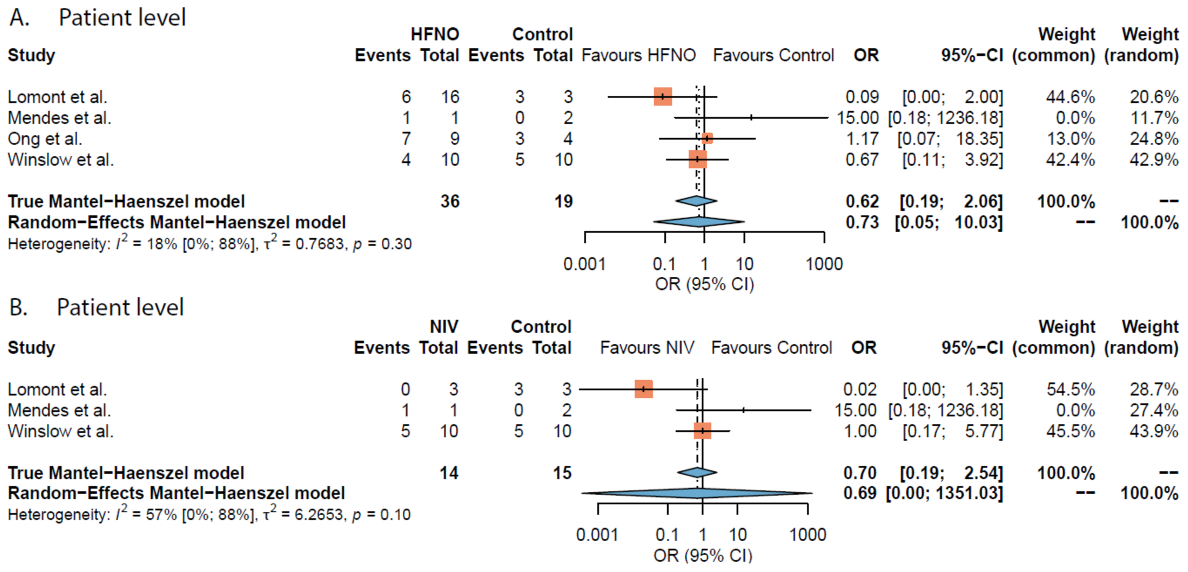

eFigure 2. Forest plots of fixed-effect and random-effects meta-analysis showing the pooled odds ratio (OR) and 95% confidence interval (95% CI) of observational studies assessing SARS-CoV-2 detection in surface samples from COVID patients treated with high flow nasal oxygen (HFNO) (panel A) and non-invasive ventilation (NIV) (panel B) versus control treatments (unsupported or conventional low flow nasal oxygen/oxygen mask).

© 2023 Zhang MX et al. *JAMA Network Open.*

eFigure 3

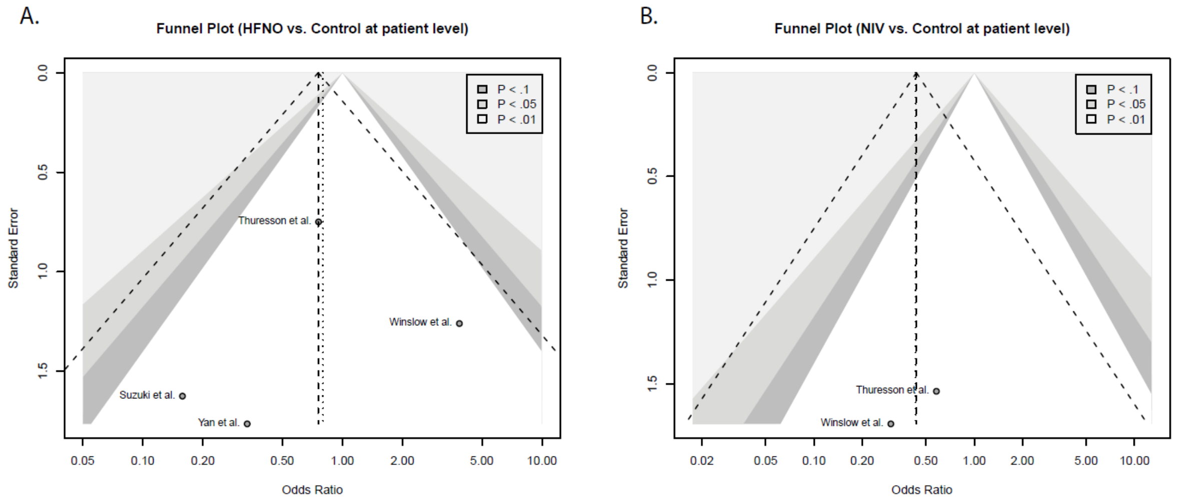

eFigure 3. Funnel plots showing the intervention high flow nasal oxygen (HFNO, panel A) and non-invasive ventilation (NIV, panel B) effect estimates for the individual studies included in the meta-analysis for detection of SARS-CoV-2 in air samples at the patient level.

© 2023 Zhang MX et al. *JAMA Network Open.*

eReferences

1. NHS. A rapid review of aerosol generating procedures (AGPs). https://www.england.nhs.uk/wp-content/uploads/2022/04/C1632_rapid-review-of-aerosol-generating-procedures.pdf. June 9, 2022.Accessed March 25, 2023.
2. Higgins JP, Altman DG, Gotzsche PC, et al. The Cochrane Collaboration's tool for assessing risk of bias in randomised trials. *BMJ*. 2011;343:d5928.
3. Wells GA, Shea B, O'Connell D, et al. The Newcastle-Ottawa Scale (NOS) for assessing the quality of nonrandomised studies in meta-analyses. Accessed April 6, 2023. http://www.ohri.ca/programs/clinical_epidemiology/oxford.asp. 2021.
4. Tufanaru C, Munn Z, Aromataris E, Campbell J, Hopp L. Chapter 3: Systematic reviews of effectiveness. In: Aromataris E, Munn Z (Editors). JBI Manual for Evidence Synthesis. JBI, Available from https://synthesismanual.jbi.globa. 2020.
5. Jermy MC, Spence CJT, Kirton R, et al. Assessment of dispersion of airborne particles of oral/nasal fluid by high flow nasal cannula therapy. *PLoS One*. 2021;16(2):e0246123.
6. Gaeckle NT, Lee J, Park Y, Kreykes G, Evans MD, Hogan CJ, Jr. Aerosol Generation from the Respiratory Tract with Various Modes of Oxygen Delivery. *Am J Respir Crit Care Med*. 2020;202(8):1115-24.
7. Leung CCH, Joynt GM, Gomersall CD, et al. Comparison of high-flow nasal cannula versus oxygen face mask for environmental bacterial contamination in critically ill pneumonia patients: a randomized controlled crossover trial. *J Hosp Infect*. 2019;101(1):84-7.
8. Miller DC, Beamer P, Billheimer D, et al. Aerosol risk with noninvasive respiratory support in patients with COVID-19. *J Am Coll Emerg Physicians Open*. 2020;1(4):521-6.
9. Bem RA, van Mourik N, Klein-Blommert R, et al. Risk of Aerosol Formation During High-Flow Nasal Cannula Treatment in Critically Ill Subjects. *Respir Care*. 2021;66(6):891-6.
10. Gall ET, Laguerre A, Noelck M, Van Meurs A, Austin JP, Foster BA. Near-field airborne particle concentrations in young children undergoing high-flow nasal cannula therapy: a pilot study. *J Hosp Infect*. 2021;113:14-21.
11. Hamada S, Tanabe N, Inoue H, Hirai T. Is High-Flow Nasal Cannula Oxygen Therapy an Aerosol-Generating Medical Procedure? *Arch Bronconeumol*. 2021;57(9):601-2.
12. Hamilton FW, Gregson FKA, Arnold DT, et al. Aerosol emission from the respiratory tract: an analysis of aerosol generation from oxygen delivery systems. *Thorax*. 2022;77(3):276-82.
13. Helgeson SA, Lee AS, Lim KG, Niven AS, Patel NM. Particulate generation with different oxygen delivery devices. *Respir Med*. 2021;181:106386.
14. Li J, Fink JB, Elshafei AA, et al. Placing a mask on COVID-19 patients during high-flow nasal cannula therapy reduces aerosol particle dispersion. *ERJ Open Res*. 2021;7(1).
15. McGain F, Humphries RS, Lee JH, et al. Aerosol generation related to respiratory interventions and the effectiveness of a personal ventilation hood. *Crit Care Resusc*. 2020;22(3):212-20.
16. Pearce E, Campen MJ, Baca JT, et al. Aerosol generation with various approaches to oxygenation in healthy volunteers in the emergency department. *J Am Coll Emerg Physicians Open*. 2021;2(2):e12390.
17. Ramsey ME, Faugno AJ, Puryear WB, et al. Characterization of SARS-CoV-2 Aerosols Dispersed During Noninvasive Respiratory Support of Patients With COVID-19. *Respir Care*. 2023;68(1):8-17.
18. Simonds AK, Hanak A, Chatwin M, et al. Evaluation of droplet dispersion during non-invasive ventilation, oxygen therapy, nebuliser treatment and chest physiotherapy in clinical practice: implications for management of pandemic influenza and other airborne infections. *Health Technol Assess*. 2010;14(46):131-72.
19. Strand-Amundsen R, Tronstad C, Elvebakk O, et al. Quantification of aerosol dispersal from suspected aerosol-generating procedures. *ERJ Open Res*. 2021;7(4).
20. Takazono T, Yamamoto K, Okamoto R, Morimoto S, Izumikawa K, Mukae H. Effects of surgical masks on droplet dispersion under various oxygen delivery modalities. *Crit Care*. 2021;25(1):89.
21. Wilson NM, Marks GB, Eckhardt A, et al. The effect of respiratory activity, non-invasive respiratory support and facemasks on aerosol generation and its relevance to COVID-19. *Anaesthesia*. 2021;76(11):1465-74.
22. Lomont A, Boubaya M, Khamis W, et al. Environmental contamination related to SARS-CoV-2 in ICU patients. *ERJ Open Res*. 2020;6(4).
23. Mendes M, Andrade Oliveira A, Pires O, et al. Sampling Methods and Risk Stratification Regarding Environmental Contamination by SARS-CoV-2. *Acta Med Port*. 2021;34(12):851-6.

© 2023 Zhang MX et al. *JAMA Network Open.*

24. Ong SWX, Lee PH, Tan YK, et al. Environmental contamination in a coronavirus disease 2019 (COVID-19) intensive care unit-What is the risk? *Infect Control Hosp Epidemiol*. 2021;42(6):669-77.
25. Suzuki T, Morioka S, Yamamoto K, et al. Nasopharyngeal SARS-CoV-2 may not be dispersed by a high-flow nasal cannula. *Sci Rep*. 2023;13(1):2669.
26. Thuresson S, Fraenkel CJ, Sasinovich S, et al. Airborne Severe Acute Respiratory Syndrome Coronavirus 2 (SARS-CoV-2) in Hospitals: Effects of Aerosol-Generating Procedures, HEPA-Filtration Units, Patient Viral Load, and Physical Distance. *Clin Infect Dis*. 2022;75(1):e89-e96.
27. Winslow RL, Zhou J, Windle EF, et al. SARS-CoV-2 environmental contamination from hospitalised patients with COVID-19 receiving aerosol-generating procedures. *Thorax*. 2022;77(3):259-67.
28. Yan K, Lin J, Albaugh S, et al. Measuring SARS-CoV-2 aerosolization in rooms of hospitalized patients. *Laryngoscope Investig Otolaryngol*. 2022;7(4):1033-41.
29. Bhaumik DK, Amatya A, Normand SL, et al. Meta-Analysis of Rare Binary Adverse Event Data. *J Am Stat Assoc*. 2012;107(498):555-67.
30. Knapp G, Hartung J. Improved tests for a random effects meta-regression with a single covariate. *Stat Med*. 2003;22(17):2693-710.
31. Efthimiou O. Practical guide to the meta-analysis of rare events. *Evid Based Ment Health*. 2018;21(2):72-6.

© 2023 Zhang MX et al. *JAMA Network Open.*

eTable 1. Main characteristics and number of identified studies

Intervention	Study design	Subjects	Main outcome	Number of studies identified reference number
HFNO	Experimental, cross-over	Patients (gram-negative pneumonia)	Positive bacterial air and surface samples	**1** [7]
	Quasi-experimental, cross-over	Patients (COVID)	Aerosol particle concentration	**1** [14]
	Observational	Patients (COVID and non-COVID)	Aerosol particle concentration	**1** [9]
	Observational	Patients (COVID)	Positive SARS-CoV-2 air samples	**5** [17, 25-28]
	Observational	Patients (COVID)	Positive SARS-CoV-2 surface samples	**4** [22-24, 27]
	(Quasi-) experimental, cross-over	Healthy adults	Aerosol particle concentration	**13** [5, 6, 8, 9, 11-13, 15-17, 19-21]
	(Quasi-) experimental, cross-over	Healthy children	Aerosol particle concentration	**1** [10]
NIV	Quasi-experimental, cross-over	Patients (upper and lower respiratory disease)	Aerosol particle concentration	**1** [18]
	Observational	Patients (COVID)	Positive SARS-CoV-2 air samples	**2** [26, 27]
	Observational	Patients (COVID)	Positive SARS-CoV-2 surface samples	**3** [22, 23, 27]
	(Quasi-) experimental, cross-over	Healthy adults	Aerosol particle concentration	**9** [6, 8, 12, 15-19, 21]

Note that some studies investigate both high flow nasal oxygen (HFNO) and non-invasive ventilation (NIV), and some studies had more than one study design.

eTable 2. Reported room conditions

Study	Room conditions			
	Pressure	Air exchange	Relative humidity	Temperature
**Bem et al.**[9]<br>Healthy subjects studies:<br>Patients studies:	normal atmospheric pressure<br>negative pressure up to –7.5 kPa		45±3%	20.5±0.5ºC
**Gaeckle et al.**[6]	negative pressure	15/hr		Set at: 21.7ºC
**Gall et al.**[10]		8.4 and 11.0/hr	Measured continuously:<br>46-66%	Measured continuously:<br>19-24.2 ºC
**Hamada et al.**[11]				
**Hamilton et al.**[12]<br>Healthy subject studies:<br>Patients studies:	 negative pressure	500-650/hr	40-60%	Set at: 20 ºC
**Helgeson et al.**[13]	negative pressure	12.9/hr		
**Jermy et al.**[5]				
**Leung et al**.[7]		6 or 12/hr		
**Li et al.**[14]	negative pressure of -0.0025kPa	12/hr		
**Lomont et al.** [22]	standard pressure			
**McGain et al.**[15]		6/hr		
**Mendes et al.**[23]	standard pressure	3/hr		
**Miller et al.**[8]	standard pressure			
**Suzuki et al.**[25]	negative pressure		56%	24.0°C
**Ong et al.**[24]				

© 2023 Zhang MX et al. *JAMA Network Open.*

**Pearce et al.**[16]	positive pressure	6/hr		
**Ramsey et al.**[17]<br><br>Healthy subjects studies:<br><br>Patient studies:		>10/hr (but subjects shielded)<br><br>4-6/hr	50-65%	21-24ºC
**Simonds et al.**[18]				
**Strand-Amundsen et al.**[19]			47.1±5.6%	26.0±0.7ºC
**Takazono et al.**[20]				
**Thuresson et al.**[26]		3-8/hr		
**Wilson et al.**[21]				
**Winslow et al.**[27]	standard and negative pressure	4-10/hr	22-58% (note differences between groups)	18-25ºC (note differences between groups)
**Yan et al.**[28]	negative pressure			

© 2023 Zhang MX et al. *JAMA Network Open.*

eTable 3. Studies on HFNO (7 pages)
A. (Quasi-)Experimental studies in patients p.1
B. Observational studies in patients p.2
C. (Quasi-)Experimental studies in healthy volunteers p.4

A. (Quasi-)Experimental studies in patients

**Study** (reference)	**Design**	**Patient category**	**Intervention**	**Control**	**Outcome(s)**	**Methodology of measurement(s)**	**Main finding(s)**
**Leung et al.**[1]<br><br>*China*	Cross-over, randomized	ICU admitted adults with gram-negative bacterial pneumonia (n=19, mean age 59 years, 50% female)	HFNO	Oxygen mask	Gram-negative bacterial and total bacterial count	Bacterial culturing (5 days) of air and surface samples at >1m and 0.4-1.5m distance respectively	HFNO does not increase viable gram-negative bacterial air or surface contamination.<br>In none of the air or surface samples from either HFNO or control condition viable gram-negative bacteria were cultured.
**Li et al.**[2]<br><br>*USA*	Cross-over (sub-analysis)	Adults with COVID (n=5)	HFNO	LFNO	- Number of particles (per m^3)<br>- Particle size distribution (µm)	Aerosol particle counter (optical, 0.3-10µm) at 30-90cm distance	HFNO does not increase production of aerosols as compared to control. For 0.5-1µm particles: mean (SD) 2744 (1317) for HFNO vs 2821 (1464) for LFNO (p=ns).

n: number of patients; ICU: intensive care unit; H/LFNO: high-/low flow nasal oxygen; ns: non-significant

© 2023 Zhang MX et al. *JAMA Network Open.*

B. Observational studies in patients

**Study** (reference) *Country*	**Patient category**	**Support modality (n)**	**Control group (n)**	**Outcome(s)**	**Methodology of measurement(s)**	**Main finding(s)**
**Bem et al.[3]**<br><br>*Netherlands*	ICU admitted adults with respiratory illness (COVID and non-COVID)	HFNO (median 50L/min) (n=10)<br><br>Mean age 70 years, 50% male	LFNO or NRM (median 7L/min) (n=7)<br>Mean age 52 years, 71,4% male	Number of particles 0.5 and 5.0 µm	Aerosol particle counter (optical, 0.5 and 5.0 µm) at 30cm and 100cm distance	HFNO is not associated with increased aerosol production for both sizes and distances.<br>For 0.5 and 5.0µm particles: median (IQR) 93.4 (59.8–130.4) and 6.8 (3.1–11.8) for HFNO vs 103.8 (100.8–107.5) and 6.0 (4.5-12.1) for control (p = ns).
**Lomont et al.[4]**<br><br>*France*	ICU admitted adults with COVID, all within 48hr of confirmed viral shedding	HFNO (n=16)	Oxygen mask (n=3)	- Positive SARS-CoV-2 surface sample (no Ct cut-off reported)	- Viral load (RT-PCR) measurement of multiple surface samples at > 1m distance	HFNO is not associated with increased detection of SARS-CoV-2 in surface samples as compared to control.<br>6/16 for HFNO patients vs 3/3 for control patients
**Mendes et al.[5]**<br><br>*Portugal*	Adults with COVID	HFNO (n=1)	Unsupported breathing (n=1) or oxygen mask (n=1)	Positive SARS-CoV-2 surface sample (no Ct cut-off reported)	Viral load (RT-PCR) measurement of surface samples at 0-2m distance	There were 2/10 surface samples positive for the single HFNO patient vs 0/10 and 0/10 for the two control patients.
**Ong et al.[6]**<br><br>*Singapore*	ICU admitted adults with COVID, all with active viral shedding	HFNO (n=9)	Unsupported breathing (n=4)	- Positive SARS-CoV-2 surface sample (no Ct cut-off reported)<br>- Positive viral culture (viable virus) up to 28 days	- Viral load (RT-PCR) measurement of multiple surface samples<br>- Viral culture of positive samples on Vero C1008 cells	HFNO is not associated with increased detection of SARS-CoV-2 in surface samples as compared to control.<br>7/9 for HFNO patients vs 3/4 for control patients

© 2023 Zhang MX et al. *JAMA Network Open.*

**Ramsey et al.**[7]<br><br>*USA*	Adults with COVID patients	HFNO (n=23, includes 8/23 confirmed active viral shedding)	Unsupported breathing (n=5, includes 3/5 confirmed active viral shedding)	Positive SARS-CoV-2 air sample (Ct <45).	Viral load (RT-PCR) measurement of multiple air samples at 30cm-3m distance.<br>Air sampling pumps (flow range 5-90L/min) collecting samples for 20-90min onto polytetrafluoroethylene or gelatin filters.	HFNO is not associated with increased detection of SARS-CoV-2 in air samples as compared to control.<br>All 49 air samples negative (only 4/7 samples collected directly at mouth by scavenger mask were positive for SARS-CoV-2: 3 with HFNO, 1 control).
**Suzuki et al.**[8]<br><br>*Japan*	Adults (median age 59 years, 65% male) with COVID, within 10 days of disease all with active viral shedding	HFNO (n=9)	LFNO (n=9)	- Positive SARS-CoV-2 air sample<br>- Positive viral culture (viable virus) on day 5.	- Viral load (RT-PCR) measurement of air samples at 0.5 and 3m distance. Air sampling pumps (flow 125L/min) collecting samples for 16min onto gelatin filters.<br>- Viral culture of positive samples on Vero E6 cells	HFNO is not associated with increased detection of SARS-CoV-2 in air samples as compared to control.<br>3/10 positive for HFNO patients vs 1/10 positive for control patients (note: one patient was sampled both during HFNO and LFNO).<br>No viral cultures of air samples were positive.
**Thuresson et al.**[9][1]<br><br>*Sweden*	Emergency/ward or ICU admitted adults (median age 59 years, majority male) with COVID	HFNO (n=30)	No HFNO or other potential AGP (n=47)	Positive SARS-CoV-2 air sample (Ct < 40), number of copies per m^3 air	Viral load (RT-PCR) measurement of multiple air samples at <1-4m distance. Liquid cyclone air sampling (flow 200L/min) for 10min.	HFNO is not associated with increased odds for positive SARS-CoV-2 air sample in adjusted analysis (e.g. for viral load, room ventilation, distance). Reported OR (CI 95%): 0.62 (0.17-2.26) and adjusted (distance/ventilation) OR (CI 95%) 0.9 (0.28-2.94). There were 3/30 HFNO patients positive vs 6/47 control patients.[1]
**Winslow et al.**[10]<br><br>*UK*	Adults (mean age 56 years, 57% male) with COVID	HFNO (n=10, with 5/10 confirmed active viral shedding)	Oxygen mask (n=10, with 9/10 confirmed active viral shedding)	- Suspected) Positive SARS-CoV-2 air/surface sample (Ct < 45 for one	- Viral load (RT-PCR) measurement of multiple air and surface samples at 50cm-2m distance. Liquid cyclone air	HFNO is not associated with increased air or surface contamination.<br>Positive air sample in 3/10 for patients with HFNO "on" and 1/10 for control patients.

				of two gene targets)<br>- positive viral culture (viable virus) (log increase in copy numbers after 5-7 days)	sampling (flow 300L/min) for 10min<br>- Viral culture of positive samples on Vero E6 cells	Positive surface sample in 5/30 samples for both HFNO and control surface samples (4/10 HFNO patients vs 5/10 control patients).<br>No statistical differences upon ANOVA and pairwise post hoc comparisons.<br>Note. no viable virus was found in air or surface samples.
**Yan et al.**[11]<br><br>*USA*	Adults (median age 57 years) with COVID	HFNO (n=4)	LFNO or unsupported breathing (n=5)	Positive SARS-CoV-2 air sample (Ct ≤ 40)	Viral load (RT-PCR) measurement of air samples at 1 and 4m distance. Air sampling pump (flow >50L/min) for ~ 3 days.	No positive air samples in HFNO patients vs 1 in the control patients.

n: number of patients; ICU: intensive care unit; AGP: aerosol-generating procedure; NRM: non-rebreather mask; H/LFNO: high-/low flow nasal oxygen; RT-PCR: real-time polymerase chain reaction. [1] data obtained from authors upon request

C. (Quasi-)Experimental studies in healthy volunteers

**Study** (reference)	**Design**	**Number of participants**	**Support modality**	**Control**	**Outcome(s)**	**Methodology of measurement**	**Main finding(s)**
**Bem et al.**[3]<br><br>*Netherlands*	Cross-over, fixed order	3 adults	HFNO (60L/min)	- Unsupported breathing<br>- NRM (15L/min)	Light pixels $x10^{-5}$ /mm^2	Laser light scattering with high-speed camera imaging.	HFNO does not increase production of aerosol particles as compared to controls. Mean (SD) 0.7 (0.6) for HFNO vs 0.6 (0.6) and 1.9 (2.7) for controls (p = ns).[1]<br>Note: coughing maneuver caused substantial increase in number of

							aerosol/droplets, independent of HFNO, but not quantified.
**Gaeckle et al.**[12]<br><br>*USA*	Cross-over, randomized	10 adults (median age 35 years, 40% female)	HFNO (10, 30 and 50L/min)	- Unsupported breathing<br>- LFNO (4L/min)<br>- Oxygen Mask (15L/min)	- Number of particles (per cm^3)<br>- Particle size distribution (µm)	Aerosol particle counter (aerodynamic, range 0.37-20µm) in closed funnel at 5 cm from mouth.	HFNO does not increase production of aerosol particles as compared to controls. Median (IQR) 0.041 (0.025–0.056) for HFNO vs 0.068 (0.046–0.091), 0.06 (0.044–0.065), 0.059 (0.055–0.074) and for controls (p = ns). Geometric mean diameter well below <5µm (range 1.1-1.6µm). Note: coughing maneuver caused significant increase in number of aerosol particles independent of HFNO.
**Gall et al.**[13]<br><br>*USA*	Cross-over, fixed order	6 children (median age 9 months)	HFNO (2ml/kg/min, max 25L/min)	Unsupported breathing	- (Change in) number of particles (per cm^3) in 0.3-10µm range, (close compared to far distance room background)	Multiple aerosol particle counters (optical, scanning mobility and condensation, 0.01-10µm) at 0.5m as compared to 2m distance	HFNO does not increase production of aerosol particles as compared to control. No mean/median values reported.
**Hamada et al.**[14]<br><br>*Japan*	Cross-over	5 adults	HFNO (40L/min)	- Unsupported breathing<br>- LFNO (4L/min)	- Number of particles (per cm^3) subtracted from background<br>- Particle size distribution (µm)	Light scattering with high-speed camera imaging and aerosol particle counter (aerodynamic, 0.5-20 µm) inside closed	HFNO does not increase production of aerosol particles as compared to controls. For <1 and >1µm particles: median (IQR) 26.1 (17.85–49.05), −1.3 (−2.55 to 0.12) for HFNO vs 28.7 (18.35–33.3), 2.9 (0.48–6.3) and 0.32 (−1.4 to 6.05), 24.7 (22.15–65.9), respectively (p = ns). Note: coughing maneuver caused significant increase in number of

						box directly at mouth	aerosol particles (independent of HFNO), but not quantified.
**Hamilton et al.**[15]<br><br>*UK*	Cross-over	25 adults (median age 35 years, 57% female	HFNO (60L/min)/	- Unsupported breathing	- Number of particles (per cm^3)<br>- Particle size distribution (µm)	Two aerosol particle counters (aerodynamic 0.5-20µm and optical 300nm-10µm) in closed funnel at 0.45m distance	HFNO does not increase production of aerosol particles compared to control, as increased HFNO-associated particles were found to be externally derived from the HFNO machine(s). Uncorrected data median (IQR): 1.861 (1.54–3.458) for HFNO vs 0.044 (0.022–0.08) for control ($p < 0.001$).
**Helgeson et al.**[16]<br><br>*USA*	Cross-over	10 adults (median age 32.5 years, 70% male)	HFNO (up to 60L/min)	- LFNO (2-10L/min<br>- Oxygen mask (2-10L/min)<br>- NRM (2-10L/min)	- Number of particles (per L per sec) subtracted from background<br>- Particle size distribution (µm)	Aerosol particle counter (optical, 0.3-10µm) at 4cm and 0.3m distance.	HFNO does not increase production of aerosol particles as compared to controls. For <5 µm particles: median (IQR) 0.0 (0.0–58.8) for HFNO vs 0.0 (0.0–0.0), 80.0 (55.8-90.3), 46.5 (19.5-96.3) for controls respectively (p = ns).
**Jermy et al.**[17]<br><br>*New Zealand*	Cross-over, randomized	10 adults (age 23-48 years, 90% male)	HFNO (30-60L/min)	Unsupported breathing	- Number of particles (pixels)<br>- Particle size and velocity<br>- Volume (µL/m^3 air) of instilled chemical marker	High-speed camera imaging (but smallest detectable 33µm) and air sampling for detection of instilled chemical marker at 10-50cm distance.	HFNO does not increase production of particles as compared to control. Only particles ≥50µm reported. Mean (min-max): 3.4 (0.0-9.9) for HFNO vs 5.0 (0.0-18.3) for control for 50µm particles during labored breathing (p = ns). HFNO results in a small increase in released oro/nasal instilled fluid (mean 6.3 vs 0µL/m^3 air), but this is 1/200 (0.5%) of the volume deposited by unsupported labored breathing or coughing/sneezing.
**McGain et al.**[18]	Cross-over, fixed order	1 adult	HFNO (30-60L/min)	- Unsupported breathing	- Number of particles (per cm^3)	Aerosol particle counters	Mean (25-75%): 0.24 (0.21-0.25) for HFNO vs 0.03 (0.01-0.04) and 0.18 (0.1-0.2) for controls. No

Australia				- Oxygen mask (15L/min)	subtracted from background<br>- Particle size distribution (µm)	(aerodynamic and single-particle mass spectrometry, 0.01-20µm) at 80cm distance	statistical analysis presented (1 subject).
**Miller et al.[19]**<br><br>*USA*	Cross-over, randomized	2 adults	HFNO (30-40L/min)	LFNO (6L/min)	- Number of particles (per m^3)<br>- Particle size distribution (µm)	Aerosol particle counter (optical, 0.3-10µm) at 0.6 and 1.8m distance.	HFNO does not increase production of aerosol particles as compared to control. Data only shown for 5-10µm particles: mean (SD) 1542 (388) for HFNO vs 1483 (636) for control for 5-10µm particles (p = ns).
**Pearce et al.[20]**<br><br>*USA*	Cross-over, fixed order	8 adults (mean age 42 years)	HFNO (15-60L/min)	Pre (unsupported) -post design, no direct comparison with NRM (15L/min)	- Number of particles (per min) in 0.15-0.3 and 0.5-2.0µm range<br>- Particle size distribution (µm)	Aerosol particle counter (optical, 0.15-7.5µm) at 0.35m distance	HFNO increased aerosol particle production (55% and 70% increase for 0.15-0.3 and 0.5-2.0µm particles vs unsupported, p <0.001), but only after the treatment (not during 10-min treatment). No absolute values reported.<br>Note. NRM led to 5% and 15% increase for 0.15-0.3 and 0.5-2.0µm particles vs unsupported.
**Ramsey et al.[7]**<br><br>*USA*	Cross-over, fixed order	5 adults	HFNO (60L/min)	Unsupported breathing	Number of particles (per cm^3)	Aerosol particle counter (optical and fluorescent bioaerosol, 0.37-30µm) at 15cm distance	HFNO does not increase production of aerosol particles as compared to control. No mean/median values reported.
**Strand-Amundsen et al.[21]**<br><br>*Norway*	Cross-over	20 adults (mean age 43 years, 65% male)	HFNO (up to 60L/min)	LFNO (4L/min)	- Number of particles (per L air), with estimation	Multiple aerosol particle counters (aerodynamic	HFNO does not increase production of aerosol particles (<5µm) as compared to control. Median (IQR): 67.8 (38.5-130.0) and 25.7 (14.9-46.0) for HFNO vs

					over time (per min)<br>- Particle size distribution (µm)	and optical, 0.3-20µm) from 30cm to 285cm distance.	60.9 (41.5-106.5) and 18.6 (14.8-24.0) for control for particles <1µm and 1-5µm respectively (p = ns). Only for >5µm particles a difference is found: 1.9 (1.2-3.5) for HFNO vs 1.2 (0.9-2.1) for control (p < 0.05).<br>Note: coughing maneuver caused significant increase in number of aerosol particles independent of HFNO.
**Takazono et al.[22]**<br><br>*Japan*	Cross-over, fixed order	5 adults	HFNO (30 and 60L/min)	- Unsupported breathing<br>- LFNO (5L/min)	- Number of particles<br>- Particle size distribution (µm)	Light scattering with high-speed camera imaging and particle counter (optical, >0.5 and >5µm) at 25-80 cm distance	HFNO does not increase production of aerosol particles as compared to controls. No mean/median values reported.<br>Note. coughing maneuver caused significant increase in number of aerosol particles independent of HFNO.
**Wilson et al.[23]**<br><br>*Australia*	Cross-over, two separate orders	10 adults (mean age 29 years, 40% female)	HFNO (20-60L/min)	Unsupported breathing	- Number of particles (per 100L)<br>- Particle size distribution (µm)<br>- Estimated particle volume (µm^3)	Aerosol particle counter (optical, 0.5-25µm) in closed cone	HFNO increases the production of aerosol particles by 2.3 fold change (CI 95% 1.2-4.4) as compared to control) with >92% particles < 5µm.<br>Note. Coughing maneuver caused significant (370.8 fold change) increase in number of aerosol particles compared to control.

H/LFNO: high-/low flow nasal oxygen; NRM: non-rebreather mask

[1] absolute data made available upon request

© 2023 Zhang MX et al. *JAMA Network Open.*

eReferences

1. Leung CCH, Joynt GM, Gomersall CD, et al. Comparison of high-flow nasal cannula versus oxygen face mask for environmental bacterial contamination in critically ill pneumonia patients: a randomized controlled crossover trial. *J Hosp Infect*. 2019;101(1):84-7.
2. Li J, Fink JB, Elshafei AA, et al. Placing a mask on COVID-19 patients during high-flow nasal cannula therapy reduces aerosol particle dispersion. *ERJ Open Res*. 2021;7(1).
3. Bem RA, van Mourik N, Klein-Blommert R, et al. Risk of Aerosol Formation During High-Flow Nasal Cannula Treatment in Critically Ill Subjects. *Respir Care*. 2021;66(6):891-6.
4. Lomont A, Boubaya M, Khamis W, et al. Environmental contamination related to SARS-CoV-2 in ICU patients. *ERJ Open Res*. 2020;6(4).
5. Mendes M, Andrade Oliveira A, Pires O, et al. Sampling Methods and Risk Stratification Regarding Environmental Contamination by SARS-CoV-2. *Acta Med Port*. 2021;34(12):851-6.
6. Ong SWX, Lee PH, Tan YK, et al. Environmental contamination in a coronavirus disease 2019 (COVID-19) intensive care unit-What is the risk? *Infect Control Hosp Epidemiol*. 2021;42(6):669-77.
7. Ramsey ME, Faugno AJ, Puryear WB, et al. Characterization of SARS-CoV-2 Aerosols Dispersed During Noninvasive Respiratory Support of Patients With COVID-19. *Respir Care*. 2023;68(1):8-17.
8. Suzuki T, Morioka S, Yamamoto K, et al. Nasopharyngeal SARS-CoV-2 may not be dispersed by a high-flow nasal cannula. *Sci Rep*. 2023;13(1):2669.
9. Thuresson S, Fraenkel CJ, Sasinovich S, et al. Airborne Severe Acute Respiratory Syndrome Coronavirus 2 (SARS-CoV-2) in Hospitals: Effects of Aerosol-Generating Procedures, HEPA-Filtration Units, Patient Viral Load, and Physical Distance. *Clin Infect Dis*. 2022;75(1):e89-e96.
10. Winslow RL, Zhou J, Windle EF, et al. SARS-CoV-2 environmental contamination from hospitalised patients with COVID-19 receiving aerosol-generating procedures. *Thorax*. 2022;77(3):259-67.
11. Yan K, Lin J, Albaugh S, et al. Measuring SARS-CoV-2 aerosolization in rooms of hospitalized patients. *Laryngoscope Investig Otolaryngol*. 2022;7(4):1033-41.
12. Gaeckle NT, Lee J, Park Y, Kreykes G, Evans MD, Hogan CJ, Jr. Aerosol Generation from the Respiratory Tract with Various Modes of Oxygen Delivery. *Am J Respir Crit Care Med*. 2020;202(8):1115-24.

© 2023 Zhang MX et al. *JAMA Network Open.*

13. Gall ET, Laguerre A, Noelck M, Van Meurs A, Austin JP, Foster BA. Near-field airborne particle concentrations in young children undergoing high-flow nasal cannula therapy: a pilot study. *J Hosp Infect*. 2021;113:14-21.
14. Hamada S, Tanabe N, Inoue H, Hirai T. Is High-Flow Nasal Cannula Oxygen Therapy an Aerosol-Generating Medical Procedure? *Arch Bronconeumol*. 2021;57(9):601-2.
15. Hamilton FW, Gregson FKA, Arnold DT, et al. Aerosol emission from the respiratory tract: an analysis of aerosol generation from oxygen delivery systems. *Thorax*. 2022;77(3):276-82.
16. Helgeson SA, Lee AS, Lim KG, Niven AS, Patel NM. Particulate generation with different oxygen delivery devices. *Respir Med*. 2021;181:106386.
17. Jermy MC, Spence CJT, Kirton R, et al. Assessment of dispersion of airborne particles of oral/nasal fluid by high flow nasal cannula therapy. *PLoS One*. 2021;16(2):e0246123.
18. McGain F, Humphries RS, Lee JH, et al. Aerosol generation related to respiratory interventions and the effectiveness of a personal ventilation hood. *Crit Care Resusc*. 2020;22(3):212-20.
19. Miller DC, Beamer P, Billheimer D, et al. Aerosol risk with noninvasive respiratory support in patients with COVID-19. *J Am Coll Emerg Physicians Open*. 2020;1(4):521-6.
20. Pearce E, Campen MJ, Baca JT, et al. Aerosol generation with various approaches to oxygenation in healthy volunteers in the emergency department. *J Am Coll Emerg Physicians Open*. 2021;2(2):e12390.
21. Strand-Amundsen R, Tronstad C, Elvebakk O, et al. Quantification of aerosol dispersal from suspected aerosol-generating procedures. *ERJ Open Res*. 2021;7(4).
22. Takazono T, Yamamoto K, Okamoto R, Morimoto S, Izumikawa K, Mukae H. Effects of surgical masks on droplet dispersion under various oxygen delivery modalities. *Crit Care*. 2021;25(1):89.
23. Wilson NM, Marks GB, Eckhardt A, et al. The effect of respiratory activity, non-invasive respiratory support and facemasks on aerosol generation and its relevance to COVID-19. *Anaesthesia*. 2021;76(11):1465-74.

© 2023 Zhang MX et al. *JAMA Network Open.*

eTable 4. Studies on NIV (4 pages)

A. (Quasi-)Experimental studies in patients p.1
B. Observational studies in patients p.2
C. (Quasi-)Experimental studies in healthy volunteers p.3

A. (Quasi-)Experimental studies in patients

**Study** (reference)	**Design**	**Patient category**	**Intervention**	**Control**	**Outcome(s)**	**Methodology of measurement(s)**	**Main finding(s)**
**Simonds et al.**[1]<br><br>*UK*	Cross-over, fixed order	Coryza (n=10), acute-on-chronic respiratory disease (n=9)	NIV (vented and non-vented mask)	- Unsupported breathing and oxygen mask for coryza patients<br>- Oxygen mask for patients with acute respiratory disease	- Number of particles (per m^3)<br>- Particle size distribution (µm)	Aerosol particle counter (optical, 0.3-10µm) at 20cm and 1m distance	NIV by vented mask increased particles (>3µm). For 10µm particles: mean difference 0.807 for coryza and 0.666 for respiratory patients vs baseline unsupported (p<0.05). No differences by non-vented NIV. No absolute mean/median values provided.

NIV: non-invasive ventilation

© 2023 Zhang MX et al. *JAMA Network Open.*

B. Observational studies in patients

**Study** (reference)	**Patient category**	**Support modality (n)**	**Control group (n)**	**Outcome(s)**	**Methodology of measurement(s)**	**Main finding(s)**
**Lomont et al.**[2]<br><br>*France*	ICU admitted adults with COVID, all within 48hr of confirmed viral shedding	NIV (n=3)	Oxygen mask (n=3)	- Positive SARS-CoV-2 surface sample (no Ct cut-off reported)	- Viral load (RT-PCR) measurement of multiple surface samples at > 1m distance	NIV is not associated with increased detection of SARS-CoV-2 in surface samples as compared to control.<br>Positive surface sample in 0/3 NIV patients vs 3/3 for control patients.
**Mendes et al.**[3]<br><br>*Portugal*	Adults with COVID	NIV (n=1)	Unsupported breathing (n=1) or oxygen mask (n=1)	Positive SARS-CoV-2 sample (no Ct cut-off reported)	Viral load (RT-PCR) measurement of surface samples at 0-2m distance	There were 4/10 surface samples positive for the single NIV patient vs 0/10 and 0/10 for the two control patients.
**Thuresson et al.**[41]<br><br>*Sweden*	Emergency/ward or ICU admitted adults (median age 59 years, majority male) with COVID	NIV (n=5)	No NIV or other potential AGP (n=47)	Positive SARS-CoV-2 air sample (Ct < 40), number of copies per m^3 air	Viral load (RT-PCR) measurement of multiple air samples at <1-4m distance. . Liquid cyclone air sampling (flow 200L/min) for 10min.	NIV is not associated with increased odds for positive SARS-CoV-2 air sample (none of the 7 air samples from 5 NIV patients were positive).
**Winslow et al.**[5]<br><br>*UK*	Adults (mean age 56 years, 57% male) with COVID	CPAP (n=10, with 8/10 confirmed active viral shedding)	Oxygen mask (n=10, with 9/10 confirmed active viral shedding)	- (Suspected) Positive SARS-CoV-2 air/surface sample (Ct < 45 for one of two gene targets)<br>- positive viral culture	- Viral load (RT-PCR) measurement of multiple air and surface samples at 50cm-2m distance. Liquid cyclone air sampling (flow 300L/min) for 10min.	CPAP is not associated with increased air or surface contamination.<br>Positive air sample in 0/10 for patients with CPAP “on” and 1/10 for control patients.<br>Positive surface sample in 6/30 samples for CPAP and 5/30 control samples (5/10 CPAP patients vs 5/10 control patients).<br>No statistical differences upon ANOVA and pairwise post hoc comparisons.

© 2023 Zhang MX et al. *JAMA Network Open.*

				(viable virus) (log increase in copy numbers after 5-7 days)	- Viral culture of positive samples on Vero E6 cells	Note. no viable virus was found in air or surface samples.

n: number of patients; ICU: intensive care unit; AGP: aerosol-generating procedure; NIV: non-invasive ventilation.
[1] absolute data made available by authors upon request

C. (Quasi-)Experimental studies in healthy volunteers

**Study** (reference)	**Design**	**Number of participants**	**Support modality**	**Control (n)**	**Outcome(s)**	**Methodology of measurement(s)**	**Main finding(s)**
**Gaeckle et al.[6]**<br><br>*USA*	Cross-over, randomized	10 adults (median age 35 years, 40% female)	NIV (bilevel positive pressure, up to 20cmH$_2$O) with vented face mask	- Unsupported breathing<br>- LFNO (4L/min)<br>- Oxygen Mask (15L/min)	- Number of particles (per cm^3)<br>- Particle size distribution (µm)	Aerosol particle counter (aerodynamic, range 0.37-20µm) in closed funnel at 5 cm from mouth.	NIV does not increase production of aerosol particles as compared to controls. Median (IQR) 0.057 (0.037–0.090) for NIV (20/10 cmH$_2$O) vs 0.068 (0.046–0.091), 0.06 (0.044–0.065), 0.059 (0.055–0.074) for controls (p = ns).<br>Note: coughing maneuver caused significant increase in number of aerosol particles independent of NIV.
**Hamilton et al.[7]**<br><br>*UK*	Cross-over	25 adults (median age 35 years, 57% female	CPAP (20cmH$_2$O) with non-vented face mask	- Unsupported breathing	- Number of particles (per cm^3)<br>- Particle size distribution (µm)	Two aerosol particle counters (aerodynamic 0.5-20µm and optical 0.3-10µm) in closed funnel at 0.45m distance	CPAP does not increase production of aerosol particles as compared to control. Median (IQR): 0.013 (0.009–0.024) for CPAP vs 0.044 (0.022–0.08) for control (p < 0.001).<br>Note: coughing maneuver caused significant increase in number of aerosol particles independent of NIV.

**McGain et al.**[8]<br><br>*Australia*	Cross-over, fixed order	1 adult	NIV (bilevel positive pressure up to 15cmH_2O) face mask	- Unsupported breathing<br>- Oxygen mask (15L/min)	- Number of particles (per cm^3) subtracted from background<br>- Particle size distribution (µm)	Aerosol particle counters (aerodynamic and scanning mobility, 0.01-20µm) at 80cm distance	NIV increases the production of aerosol particles.<br>Mean (25-75%): 29.7 (25.7-35.2) for NIV vs 0.03 (0.01-0.04) and 0.18 (0.1-0.2) for controls. No statistical analysis presented (1 subject).
**Miller et al.**[9]<br><br>*USA*	Cross-over, randomized	2 adults	CPAP (5-15cmH_2O) with face mask	LFNO (6L/min)	- Number of particles (per m^3)<br>- Particle size distribution (µm)	Aerosol particle counter (optical, 0.3-10µm) at 0.6 and 1.8m distance.	CPAP does not increase production of aerosol particles as compared to control. Mean (SD): 1812 (589) for CPAP vs 2189 (827) for control for 5-10µm particles (p = ns).
**Pearce et al.**[10]<br><br>*USA*	Cross-over, fixed order	8 adults (mean age 42 years)	CPAP (10cmH_2O) with face mask	Pre (unsupported) -post design, no direct comparison with NRM (15L/min)	- Number of particles (per min) in 0.15-0.3 and 0.5-2.0µm range<br>- Particle size distribution (µm)	Aerosol particle counter (optical, 0.15-7.5µm) at 0.35m distance	CPAP does not increase aerosol particle production (15% decrease for 0.15-0.3µm particles vs unsupported, p <0.001) during the treatment. No absolute values reported.
**Ramsey et al.**[11]<br><br>*USA*	Cross-over, fixed order	5 adults	NIV (bilevel positive pressure up to 12cmH_2O) with face mask	Unsupported breathing	Number of particles (per cm^3)	Aerosol particle counter (optical and fluorescent bioaerosol, 0.37-30µm) at 15cm distance	NIV does not increase production of aerosol particles as compared to control. No mean/median values reported.
**Simonds et al.**[1]<br><br>*UK*	Cross-over, fixed order	12 adults (mean age 33.7 years)	NIV (bilevel positive pressure up to 20cmH_2O)	- Unsupported<br>- Oxygen mask	- Number of particles (per m^3)	Aerosol particle counter (optical, 0.3-10µm) at	NIV does not increase production of aerosol particles as compared to baseline control. No direct

© 2023 Zhang MX et al. *JAMA Network Open.*

			with vented and non-vented face mask		- Particle size distribution (µm)	20cm and 1m distance	comparison with oxygen mask. No mean/median values provided.
**Strand-Amundsen et al.[12]**<br><br>*Norway*	Cross-over	20 adults (mean age 43 years, 65% male)	NIV (bilevel positive pressure, up to 10cmH_2O) with non-vented face mask	LFNO (4L/min)	- Number of particles (per L air), with estimation over time (per min)<br>- Particle size distribution (µm)	Multiple aerosol particle counters (aerodynamic and optical, 0.3-20µm) from 30cm to 285cm distance.	NIV does not increase production of aerosol particles as compared to control. Median (IQR): 66.4 (33.3-102.4) and 22.1 (13.3-26.4) for NIV vs 18.6 (14.8-24.0) and 60.9 (41.5-106.5) for control for particles <1µm and 1-5µm respectively (p = ns).<br>Note: coughing maneuver caused significant increase in number of aerosol particles independent of NIV.
**Wilson et al.[13]**<br><br>*Australia*	Cross-over, two separate orders	10 adults (mean age 29 years, 40% female)	NIV (bilevel positive pressure up to 25cmH_2O) with vented and non-vented face mask	Unsupported breathing	- Number of particles (per 100L)<br>- Particle size distribution (µm)<br>- Estimated particle volume (µm^3)	Aerosol particle counter (optical, 0.5-25µm) in closed cone	NIV increases the production of aerosol particles by 2.6 fold increase (CI 95% 1.7-4.1) for vented and 7.8 fold increase (CI 95% 4.4-13.6) for non-vented as compared to control with >92% particles < 5µm.<br>Note. Coughing maneuver caused significant (370.8 fold change) increase in number of aerosol particles compared to control.

n: number of patients; ICU: intensive care unit; NIV: non-invasive ventilation; CPAP: continuous positive airway pressure; NRM: non-rebreather mask; H/LFNO: high/low flow nasal oxygen; NRM: non-rebreather mask

© 2023 Zhang MX et al. *JAMA Network Open.*

eReferences

1. Simonds AK, Hanak A, Chatwin M, et al. Evaluation of droplet dispersion during non-invasive ventilation, oxygen therapy, nebuliser treatment and chest physiotherapy in clinical practice: implications for management of pandemic influenza and other airborne infections. *Health Technol Assess*. 2010;14(46):131-72.
2. Lomont A, Boubaya M, Khamis W, et al. Environmental contamination related to SARS-CoV-2 in ICU patients. *ERJ Open Res*. 2020;6(4).
3. Mendes M, Andrade Oliveira A, Pires O, et al. Sampling Methods and Risk Stratification Regarding Environmental Contamination by SARS-CoV-2. *Acta Med Port*. 2021;34(12):851-6.
4. Thuresson S, Fraenkel CJ, Sasinovich S, et al. Airborne Severe Acute Respiratory Syndrome Coronavirus 2 (SARS-CoV-2) in Hospitals: Effects of Aerosol-Generating Procedures, HEPA-Filtration Units, Patient Viral Load, and Physical Distance. *Clin Infect Dis*. 2022;75(1):e89-e96.
5. Winslow RL, Zhou J, Windle EF, et al. SARS-CoV-2 environmental contamination from hospitalised patients with COVID-19 receiving aerosol-generating procedures. *Thorax*. 2022;77(3):259-67.
6. Gaeckle NT, Lee J, Park Y, Kreykes G, Evans MD, Hogan CJ, Jr. Aerosol Generation from the Respiratory Tract with Various Modes of Oxygen Delivery. *Am J Respir Crit Care Med*. 2020;202(8):1115-24.
7. Hamilton FW, Gregson FKA, Arnold DT, et al. Aerosol emission from the respiratory tract: an analysis of aerosol generation from oxygen delivery systems. *Thorax*. 2022;77(3):276-82.
8. McGain F, Humphries RS, Lee JH, et al. Aerosol generation related to respiratory interventions and the effectiveness of a personal ventilation hood. *Crit Care Resusc*. 2020;22(3):212-20.
9. Miller DC, Beamer P, Billheimer D, et al. Aerosol risk with noninvasive respiratory support in patients with COVID-19. *J Am Coll Emerg Physicians Open*. 2020;1(4):521-6.
10. Pearce E, Campen MJ, Baca JT, et al. Aerosol generation with various approaches to oxygenation in healthy volunteers in the emergency department. *J Am Coll Emerg Physicians Open*. 2021;2(2):e12390.
11. Ramsey ME, Faugno AJ, Puryear WB, et al. Characterization of SARS-CoV-2 Aerosols Dispersed During Noninvasive Respiratory Support of Patients With COVID-19. *Respir Care*. 2023;68(1):8-17.
12. Strand-Amundsen R, Tronstad C, Elvebakk O, et al. Quantification of aerosol dispersal from suspected aerosol-generating procedures. *ERJ Open Res*. 2021;7(4).
13. Wilson NM, Marks GB, Eckhardt A, et al. The effect of respiratory activity, non-invasive respiratory support and facemasks on aerosol generation and its relevance to COVID-19. *Anaesthesia*. 2021;76(11):1465-74.

© 2023 Zhang MX et al. *JAMA Network Open.*

eTable 5. Summary of sensitivity analyses for HFNO and NIV studies

Case type	Analysis	Model type	Summary measure	Effect estimate (95% CI)
HFNO				
Sample level:	Sensitivity 1	True MH[a]	OR	0.69 (0.25 to 1.88)
	Sensitivity 2	Random-effects[b]	ASD	-0.10 (-0.42 to 0.21)[c]
Patient level:	Sensitivity 3	True MH[a]	OR	0.76 (0.26 to 2.22)
	Sensitivity 4	Random-effects[b]	ASD	-0.11 (-0.48 to 0.26)[c]
NIV				
Sample level:	Sensitivity 1	True MH[a]	OR	0.38 (0.04 to 3.57)
Patient level:	Sensitivity 2	True MH[a]	OR	0.44 (0.05 to 4.15)

a, fixed-effect Mantel-Haenszel model; b, random-effects model with Knapp-Hartung adjustment; c, arcsine difference < 0 favors intervention and > 0 favors control. CI, confidence interval; HFNO, High flow nasal oxygen; MH, Mantel-Haenszel; OR, odds ratio; ASD, Arcsine difference; NIV, non-invasive ventilation.

© 2023 Zhang MX et al. *JAMA Network Open.*
