# Supplementary material for: Generation of Aerosols by Noninvasive Respiratory Support Modalities: A Systematic Review and Meta-Analysis
Source: JAMA Netw Open. 2023 Oct 11;6(10):e2337258. doi: 10.1001/jamanetworkopen.2023.37258 (PMC10568354; doi:10.1001/jamanetworkopen.2023.37258)
Supplement: Supplement 2. — Data Sharing Statement [file jamanetwopen-e2337258-s002.pdf]

Data Sharing Statement

Zhang. Generation of Aerosols by Noninvasive Respiratory Support Modalities. *JAMA Netw Open*. Published October 11, 2023. doi:10.1001/jamanetworkopen.2023.37258

Data
Data available: Yes
Data types: Other (please specify)
Additional Information: Extracted data can be openly accessed upon reasonable request.
How to access data: Extracted data can be openly accessed upon reasonable request.
When available: With publication

Supporting Documents
Document types: None

Additional Information
Who can access the data: Extracted data can be openly accessed upon reasonable request.
Types of analyses: Meta-analysis
Mechanisms of data availability: with investigator support
